# A novel function for CDK2 activity at meiotic crossover sites

**DOI:** 10.1371/journal.pbio.3000903

**Published:** 2020-10-19

**Authors:** Nathan Palmer, S. Zakiah A. Talib, Priti Singh, Christine M. F. Goh, Kui Liu, John C. Schimenti, Philipp Kaldis

**Affiliations:** 1 Institute of Molecular and Cell Biology (IMCB), A*STAR (Agency for Science, Technology, and Research), Singapore, Republic of Singapore; 2 Department of Biochemistry, National University of Singapore (NUS), Singapore, Republic of Singapore; 3 Department of Biomedical Sciences, College of Veterinary Medicine, Cornell University, Ithaca, New York, United States of America; 4 Department of Obstetrics and Gynecology, Li Ka Shing Faculty of Medicine, The University of Hong Kong, Hong Kong, China; 5 Shenzhen Key Laboratory of Fertility Regulation, Center of Assisted Reproduction and Embryology, The University of Hong Kong—Shenzhen Hospital, Shenzhen, China; 6 Department of Clinical Sciences, Clinical Research Centre, Lund University, Malmö, Sweden; Stowers Institute for Medical Research, UNITED STATES

## Abstract

Genetic diversity in offspring is induced by meiotic recombination, which is initiated between homologs at >200 sites originating from meiotic double-strand breaks (DSBs). Of this initial pool, only 1–2 DSBs per homolog pair will be designated to form meiotic crossovers (COs), where reciprocal genetic exchange occurs between parental chromosomes. Cyclin-dependent kinase 2 (CDK2) is known to localize to so-called “late recombination nodules” (LRNs) marking incipient CO sites. However, the role of CDK2 kinase activity in the process of CO formation remains uncertain. Here, we describe the phenotype of 2 *Cdk2* point mutants with elevated or decreased activity, respectively. Elevated CDK2 activity was associated with increased numbers of LRN-associated proteins, including CDK2 itself and the MutL homolog 1 (MLH1) component of the MutLγ complex, but did not lead to increased numbers of COs. In contrast, reduced CDK2 activity leads to the complete absence of CO formation during meiotic prophase I. Our data suggest an important role for CDK2 in regulating MLH1 focus numbers and that the activity of this kinase is a key regulatory factor in the formation of meiotic COs.

## Abstract 2

The cyclin-dependent kinase CDK2 is essential for fertility and regulates several stages of meiosis. This study reveals that increased CDK2 activity leads to elevated numbers of meiotic crossovers, whereas lower CDK2 activity prevents crossovers, suggesting that CDK2 plays an important role in crossover formation and is a key regulator of the crossover designation process.

## Introduction

In most eukaryotic organisms, crossover (CO) is required for the proper disjunction of homologous chromosomes at the first meiotic division. Additionally, it promotes the genetic heterogeneity of gametes through the reciprocal exchange of genetic material [1]. During meiosis I prophase, interhomolog recombination is initiated via the deliberate or “programmed” induction of double-strand breaks (DSBs) by the endonuclease SPO11 (meiotic recombination protein SPO11), in conjunction with additional proteins [2–4]. In mice, 200–300 meiotic DSBs undergo “designation/selection” to become approximately 22 ± 3 meiotic COs during early meiotic prophase I. Most of the remaining sites become “deselected” from this fate and are repaired as noncrossovers (NCOs) that are nevertheless essential for proper pairing and synapsis of homologs (see review: [5]). During meiotic prophase, at least one “obligate” meiotic CO is formed per homolog pair. This concept, termed “CO assurance” [6], is essential for preventing chromosomal missegregation (nondisjunction) during the first meiotic division and the generation of aneuploid gametes. Here, COs also play a crucial structural role by acting as anchor points between homologs, allowing their biorientation along the meiotic spindle.

The mechanisms determining which early recombination events are “selected” to form COs are still not fully understood. One important determining factor in this process involves the preferential binding of specific protein complexes to maturing CO sites to form structures known as late recombination nodules (LRNs) [7, 8]. These factors have been termed “pro-CO” factors and are thought to maintain the stability of DNA intermediates destined to become CO sites. One such factor is cyclin-dependent kinase 2 (CDK2). To date, the evidence supporting the notion that CDK2 is involved in the CO formation process has been heavily based upon its localization during meiotic prophase. CDK2 can be visualized at telomeres and transiently as 1–2 interstitial foci marking LRNs along the length of fully synapsed homologs [9–16]. Although CDK2 was first proposed to function at these sites almost 2 decades ago, this topic has yet to be properly investigated.

CDK2 is a multifunctional kinase that is recognized to play multiple roles during germ cell development. These include regulating cell fate in spermatogonial stem cells [17], regulation of transcription in spermatocytes [18], and promoting homolog synapsis during meiotic prophase I ([10]; for reviews, also see [19–22]). During zygonema of meiotic prophase, CDK2 and its noncanonical activating partner Speedy A (see below) interact to promote stable interactions between telomeres and the inner nuclear envelope. This action is essential for the DSB-stimulated homology search process needed for pairing of homologous chromosomes ([23–25]; reviewed in [19]; see also [26]). Consequentially, *Cdk2*^*−/−*^ and *Speedy A*^*−/−*^ meiocytes display extensive nonhomologous synapsis and arrest at an identical pachytene-like stage [10, 27, 28]. This effectively precludes accurate interhomolog recombination, causing germ cell death because of unrepaired meiotic DSBs [10, 25].

The synaptic functions of CDK2 require its association with a member of the Speedy/RINGO (speedy inducer of meiotic maturation/rapid inducer of G2/M progression in oocytes) family of cyclin-like CDK-activating proteins, Speedy A [29–31]. Despite sharing little homology to cyclins, Speedy A, like other Speedy/RINGO proteins, binds to and activates CDK2 in a cyclin-like manner through its RINGO box domain [32, 33]. Uniquely, CDK-Speedy/RINGO protein complexes display catalytic activity in the absence of activating phosphorylation within their T-loop/activation loop domain of the CDK [34]. This activating phosphorylation is required for the catalytic activity of most CDK/cyclin complexes, which in CDK2 occurs at threonine 160 (Thr160) [35, 36]. Mutagenesis of the activating Thr160 to a nonphosphorylatable alanine residue thus prevents CDK activation by cyclins [35, 37–40]. Because the catalytic activity of CDK2/cyclin complexes, but not CDK2/Speedy A complexes, would be sensitive to the loss of T-loop phosphorylation, we hypothesized that mutagenesis of Thr160 might preserve the synaptic functions of CDK2/Speedy A while simultaneously compromising the ability of CDK2 to function at LRNs (to which CDK2 localizes independently of Speedy A). In addition to this “activation-deficient” model of CDK2/cyclin complexes, we also explore the converse situation using a mouse model in which CDK2/cyclin complexes are not subject to normal inhibitory regulation. Tyr15 (Y15) of CDK2 is an inhibitory regulatory site that is phosphorylated by the Wee1-like protein kinase, WEE1. Therefore, mutation of Y15 leads to either premature activation or elevated activity of CDK2 [17, 41].

The partial-loss-of-function (*Cdk2*^*T160A*^) and gain-of-function (*Cdk2*^*Y15S*^) CDK2 mouse models have been characterized in prior work from the Schimenti and Kaldis laboratories [15, 42]. Although both of these models result in male infertility, the meiotic defects observed are less severe than that seen upon complete ablation of *Cdk2* or its associated kinase activity, allowing us to investigate for the first time the role of this kinase in the process of meiotic CO formation.

## Results

### *Cdk2*^*T160A*^ spermatocytes exhibit incomplete prophase I progression

CDK2 and its noncanonical binding partner Speedy A are essential for the completion of the first meiotic division. The disruption of CDK2/Speedy complexes through the deletion of either *Cdk2* or *Speedy A* leads to severe synaptic defects and meiotic arrest in an early-pachytene–like arrest stage, precluding the analysis of later meiotic stages [24, 25, 27, 28]. Thus, the potential requirement for other, non-Speedy A–containing CDK2 complexes during these later meiotic stages remains uncertain.

As a first approach to investigate this point, we analyzed the meiotic progression of spermatocytes from the “partial-loss-of-function” *Cdk2*^*T160A*^ mouse [42]. To understand the extent and fidelity of meiotic progression in this mutant, we analyzed the synaptonemal complex (SC) formation throughout meiotic prophase I. This was done by immunolabeling surface spreads of *Cdk2*^*T160A*^ spermatocytes for protein components of the axial element (synaptonemal complex protein 3, SYCP3) and the transverse element (SYCP1) of the SC scaffold.

**Fig 1:**
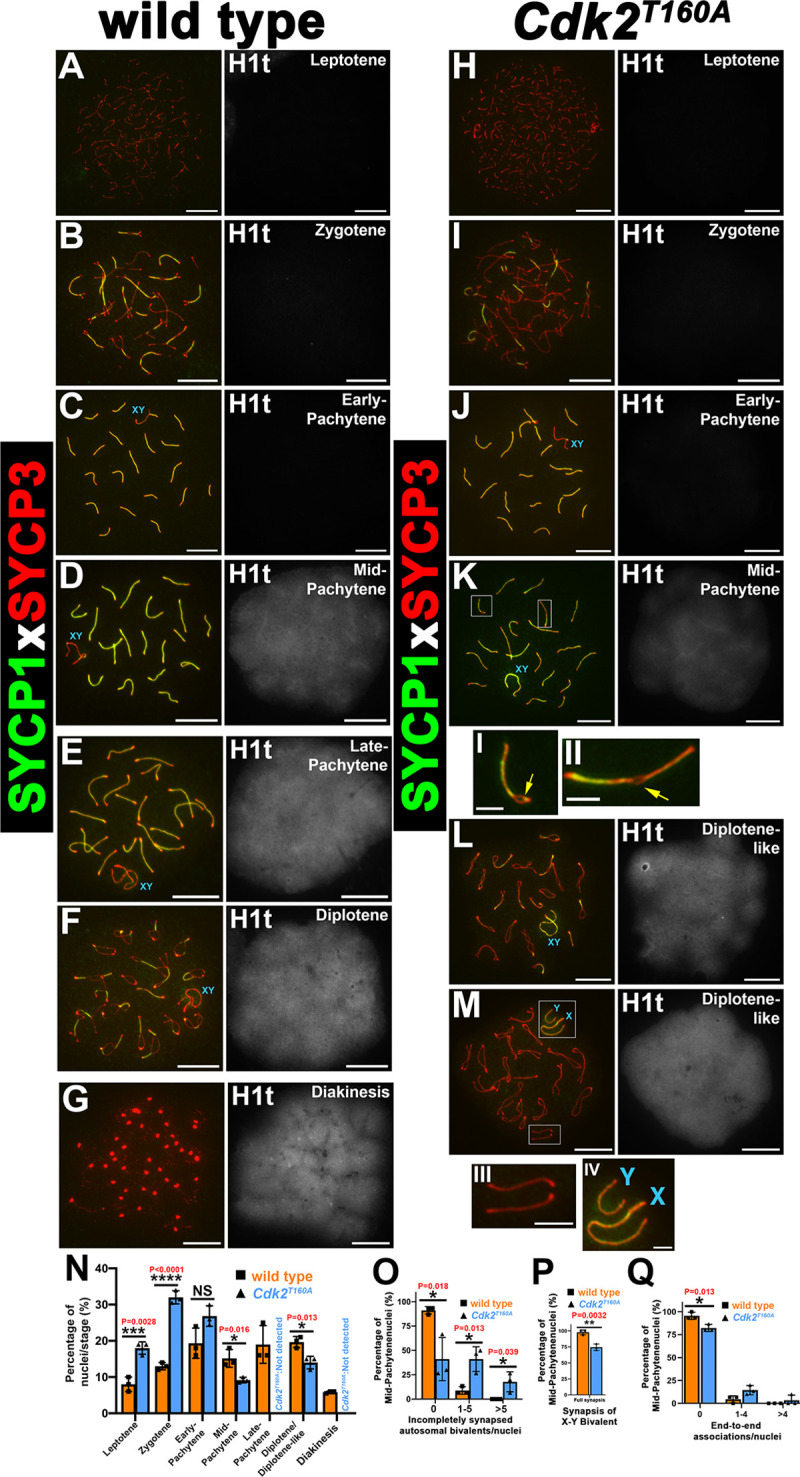
SC formation, meiotic staging analysis, and synaptic defects analysis in *Cdk2*^*T160A*^ spermatocytes. Chromosome spread preparations from adult (postnatal day 40) testes coimmunostained for SYCP1 (green) and SYCP3 (red) are shown for WT (A–G) or *Cdk2*^*T160A*^ spermatocytes (H–M) for selected stages of meiotic prophase I. For staging purposes, histone H1t (white) is shown to the right of each image. H1t positivity is indicative of mid-pachytene stage onwards. During early-pachytene, both WT (C) and *Cdk2*^*T160A*^ (J) spermatocytes show SYCP3 colocalization with SYCP1, indicating the normal formation of the SC scaffold structure. WT spermatocytes progress normally into mid-pachytene (D), late-pachytene (E), diplotene (F), and diakinesis stages (G). Mid-pachytene–stage *Cdk2*^*T160A*^ (K) spermatocytes show premature desynapsis. Yellow arrows in insets I and II highlight areas of desynapsis between homologs. Late-pachytene stage is not observed in the *Cdk2*^*T160A*^ mutant. Instead, meiosis progresses to a diplotene-like stage with desynaspsis observed between both autosomal homologs and the X–Y bivalent (L and M and insets III and IV). All main panels are representative of at least 20 images taken for specified stages. Similar staining patterns were confirmed in at least 3 biological replicates. In all main panels, scale bars are representative of 5 μm; in all inset pictures, scale bars are representative of 1.25 μm. Meiotic staging analysis (N) shows the percentages of spermatocytes observed in each stage of meiotic prophase. Asynapsis was quantified specifically for mid-pachytene stages by counting the percentages nuclei containing incompletely synapsed autosomal bivalents, classified as having incomplete colocalization of SYCP1 and SYCP3 along the entire chromosomal length (O). X–Y bivalent synapsis was quantified specifically for mid-pachytene stages by counting the percentage of nuclei containing fully synapsed X–Y chromosomes, classified as being physically associated with no clear separation (P). End-to-end associations were quantified specifically for mid-pachytene stages by counting the percentages nuclei containing bivalents that were joined end-to-end; the joining of 2 ends between distinct bivalents was quantified as a single event (Q). For N–Q, WT or *Cdk2*^*T160A*^ data are shown using orange and blue bars, respectively. Each data point is a mean percentage ± SD determined from 3 biological replicates. For each biological replicate, percentages were determined from at least 200 spermatocyte images. All data were assumed to be non-normally distributed. Statistical significance between genotypes was determined by unpaired *t* test. Significance and *P*-values are reported directly over each comparison. The underlying data for (N, O, P, Q) can be found in S1 Data. CDK2, cyclin-dependent kinase 2; SC, synaptonemal complex; SD, standard deviation; SYCP, synaptonemal complex protein; WT, wild-type.

Unlike *Cdk2*^*−/−*^ spermatocytes, synapsis in *Cdk2*^*T160A*^ spermatocytes appeared to progress normally from leptonema through early pachynema, indicating that T-loop mutagenesis was permissive of accurate homolog pairing (compare Fig 1A–1C to Fig 1H–1J). Typical configurations of each meiotic stage were observed, and SYCP1 colocalized with SYCP3 at recently synapsed chromosomal axes. This was observed in both juvenile (P15, P18) and adult (P40) mutant mice. Unless otherwise stated, all surface-spread images used in this study were prepared from the P40 time point. By early pachynema, when synapsis is complete, full colocalization of SYCP3 with SYCP1 was seen on all autosomes and also at the synapsed pseudoautosomal region (PAR) of the X and Y chromosomes (Fig 1J; compare with wild-type [WT], Fig 1C). This is in stark contrast to *Cdk2*^*−/−*^ spermatocytes, which arrest prematurely in a pachytene-like stage, as previously reported [10]. Interestingly, analysis of the various stages of prophase I revealed a disproportionately increased percentage of *Cdk2*^*T160A*^ spermatocytes in leptotene and zygotene stages (18% ± 1.4% and 32% ± 1.4%, respectively), as compared with WT (8% ± 1.6% and 13% ± 0.8%, respectively; see Fig 1N for quantification). Although the numbers of early-pachytene–stage *Cdk2*^*T160A*^ spermatocytes were not significantly different from WT, there was a marked and significant decrease in all later meiotic stages, suggesting a defect in meiotic progression through prophase I. Strikingly, only 9.2% ± 0.6% of *Cdk2*^*T160A*^ spermatocytes achieved a mid-pachytene morphology, as determined by positivity for the mid-pachytene–specific histone marker H1t (Fig 1K). Despite histone H1t positivity, the colocalization of SYCP1 and SYCP3 in these cells was less complete as compared to WT (Fig 1D). We were also unable to detect any *Cdk2*^*T160A*^ spermatocytes with late-pachytene morphology as shown for WT (Fig 1E). Nonetheless, 14% ± 1.4% *Cdk2*^*T160A*^ spermatocytes achieved what will herein be referred to as a “diplotene-like morphology.” Here, diplotene-like describes histone H1t-positive nuclei, exhibiting separation of SC axes as typically seen for WT diplotene cells (Fig 1F). Notably, the separation of SC axes in these diplotene-like cells was often accompanied by complete separation of autosomal homologs (Fig 1, inset III) and/or sex chromosomes (Fig 1, inset IV). This diplotene-like state was the last observable meiotic stage seen in *Cdk2*^*T160A*^ spermatocytes because diakinesis stage chromosomes (as shown in Fig 1G) could not be detected. The decreased number of nuclei reaching stages beyond mid-pachytene of prophase I coupled with the impaired synapsis observed at this stage, led us to suspect that meiotic arrest was occurring at this stage. To determine whether synapsis defects might be contributing towards the defective meiotic progression of *Cdk2*^*T160A*^ spermatocytes, we quantified the number of homolog pairs (bivalents) retaining full overlap of SYCP3 and SYCP1 at mid-pachytene. In this manner, we found that only 41% ± 22% *Cdk2*^*T160A*^ nuclei retain full synapsis of all homologs in mid-pachytene as compared to 91% ± 4% of WT nuclei, with 18% ± 10% of *Cdk2*^*T160A*^ nuclei displaying greater than 6 homologs showing incomplete synapsis at this stage as compared to 0% of WT nuclei (Fig 1O). Often, such incompletely synapsed chromosomes displayed small portions of SC axes that were observed to be incompletely synapsed (Fig 1, insets I, II, marked by yellow arrows). No such synaptic defect was observed in early-pachytene–stage nuclei (Fig 1J), suggesting that this loss of synapsis likely occurs after these regions were initially paired. Notably, this synapsis defect did not seem to be isolated to autosomal bivalents, as we found that 26% ± 5% of mid-pachytene *Cdk2*^*T160A*^ nuclei displayed complete asynapsis of the X–Y bivalent, whereas almost all (98% ± 4%) WT nuclei retained full synapsis at this stage (Fig 1P). Together, these results suggest that T-loop mutagenesis of CDK2 is permissive for the initial pairing of homologous chromosomes in early prophase I, but thereafter, premature desynapsis of the SC complex leads to separation of homologs and precocious entry into a diplotene-like state. The reduced numbers of spermatocytes reaching a diplotene-like stage led us to hypothesize that a significant number of spermatocytes may be lost through apoptotic arrest before the end of prophase I. The complete absence of diakinesis stage *Cdk2*^*T160A*^ spermatocytes further suggested that those cells reaching a diplotene-like state are likely abnormal and unable to complete the first meiotic division.

In addition to the synaptic defects described above, we also found that 18% ± 4% of mid-pachytene–stage *Cdk2*^*T160A*^ nuclei displayed end-to-end associations of bivalents, as compared with only 4% ± 3% of WT nuclei. In affected nuclei, typically only 1–4 homolog pairs were seen to exhibit this defect, with only 3% ± 6% of all counted *Cdk2*^*T160A*^ nuclei exhibiting greater than 4 affected homologs (Fig 1Q). Interestingly, this defect did not seem to be directly related to the completeness of synapsis observed for affected bivalents because end-to-end associations were not significantly higher in bivalents scored as having incomplete synapsis in Fig 1O.

### Meiotic arrest and apoptosis of *Cdk2*^*T160A*^ spermatocytes before metaphase I

We considered that both the premature desynapsis and end-to-end association defects could contribute towards the failure of *Cdk2*^*T160A*^ spermatocytes to complete the first meiotic division and thus sought to further characterize the arrest point of this mutant through phenotypic and histological analysis of mutant testes. In agreement with our above results, the testes of *Cdk2*^*T160A*^ animals were found to be significantly smaller than those of WT animals across several developmental time points, suggesting a defect in their normal development (S1A Fig). This difference became significant at P18 (*P* = 0.0035, WT versus *Cdk2*^*T160A*^), which roughly corresponds with the progression of spermatocytes through meiotic prophase I of the first wave of spermatogenesis. This decrease in testis/bodyweight ratio became more apparent by P30 (*P* < 0.0001, WT versus *Cdk2*^*T160A*^), by which additional waves of spermatogenesis have been initiated in WT animals (yellow box in S1A Fig). At all time points from P14 onwards, the testis/bodyweight ratio of *Cdk2*^*T160A*^ animals was significantly higher as compared to *Cdk2*^*−/−*^ animals. This supports our observation that the *Cdk2*^*T160A*^ meiotic arrest occurs at a later stage compared to *Cdk2*^*−/−*^ due to the further progression of *Cdk2*^*T160A*^ spermatocytes through meiotic prophase. To determine whether any *Cdk2*^*T160A*^ spermatocytes were able to complete meiosis I, we prepared hematoxylin–eosin (HE) stained sections from P30 WT and *Cdk2*^*T160A*^ testes. At this stage, the first meiotic division has been completed for WT germ cells within the first wave of spermatogenesis, and round as well as elongating spermatids are easily detectable in the lumen of testis sections (S1B Fig, [43]). However, we did not detect any secondary spermatocytes or postmeiotic round spermatids in *Cdk2*^*T160A*^ sections (S1D and S1E Fig; compare with WT in S1B Fig and *Cdk2*^*−/−*^ in S1C Fig).

To determine more accurately the stage at which spermatocytes are lost via apoptosis, TUNEL assays were performed on P30 testis sections from WT, *Cdk2*^*−/−*^, and *Cdk2*^*T160A*^ (S2A–S2D Fig). Following our previous observations from S1 Fig, we detected tubules from *Cdk2*^*T160A*^ mice containing both apoptotic (TUNEL-positive) and healthy (TUNEL-negative) pachytene-stage spermatocytes (identified with green P* or red P, respectively; S2B Fig and insets B1 and B2). These were found at a significantly higher frequency than in WT testes (3.5 ± 0.5 apoptotic cells/tubule in *Cdk2*^*T160A*^ as compared with 0.2 ± 0.05 apoptotic cells/tubule in WT). Such coexistence of apoptotic and healthy spermatocytes was not observed in *Cdk2*^*−/−*^ testes, whose tubules were mostly devoid of meiotic cells (S2C Fig). The remainder of tubules with spermatocytes displayed almost complete positivity for TUNEL staining (average of 1.79 ± 0.2 apoptotic cells/tubule). These results support the observation that *Cdk2*^*T160A*^ spermatocytes progress further through meiosis than *Cdk2*^*−/−*^ spermatocytes. No apoptosis could be observed in leptotene/zygotene stage cells in any of the tested genotypes (blue L/Z in S2A–S2C Fig), suggesting that the bulk of apoptosis observed in *Cdk2*^*T160A*^ spermatocytes occurs specifically at pachytene stage or later. Staging analysis of tubules containing apoptotic cells using standard cytological guidelines [44, 45] indicated that the majority of apoptotic cells could be observed in epithelial stage IV tubules, which are known to be enriched for mid-pachytene–stage spermatocytes [44, 45]. Indeed, in the majority of meiotic arrest models, apoptosis is commonly observed at this stage [46, 47].

Our results suggest that the majority of *Cdk2*^*T160A*^ spermatocytes progress normally to an early-pachytene stage but thereafter progress abnormally from mid-pachytene stage onwards. Subsequent mid-pachytene–stage *Cdk2*^*T160A*^ spermatocytes display phenotypic abnormalities including a loss of SC component colocalization, premature desynapsis of paired axes, and end-to-end association of bivalents. The decreased observance or absence of diplotene/diakinesis stage spermatocytes in this model, in addition to our TUNEL staining analyses, suggests an apoptotic loss of spermatocytes before the prophase I to metaphase I transition because the meiotic arrest in *Cdk2*^*T160A*^ spermatocytes prevents the first meiotic division.

### Analysis of SC defects in mid-pachytene–stage *Cdk2*^*T160A*^ spermatocytes

**Fig 2:**
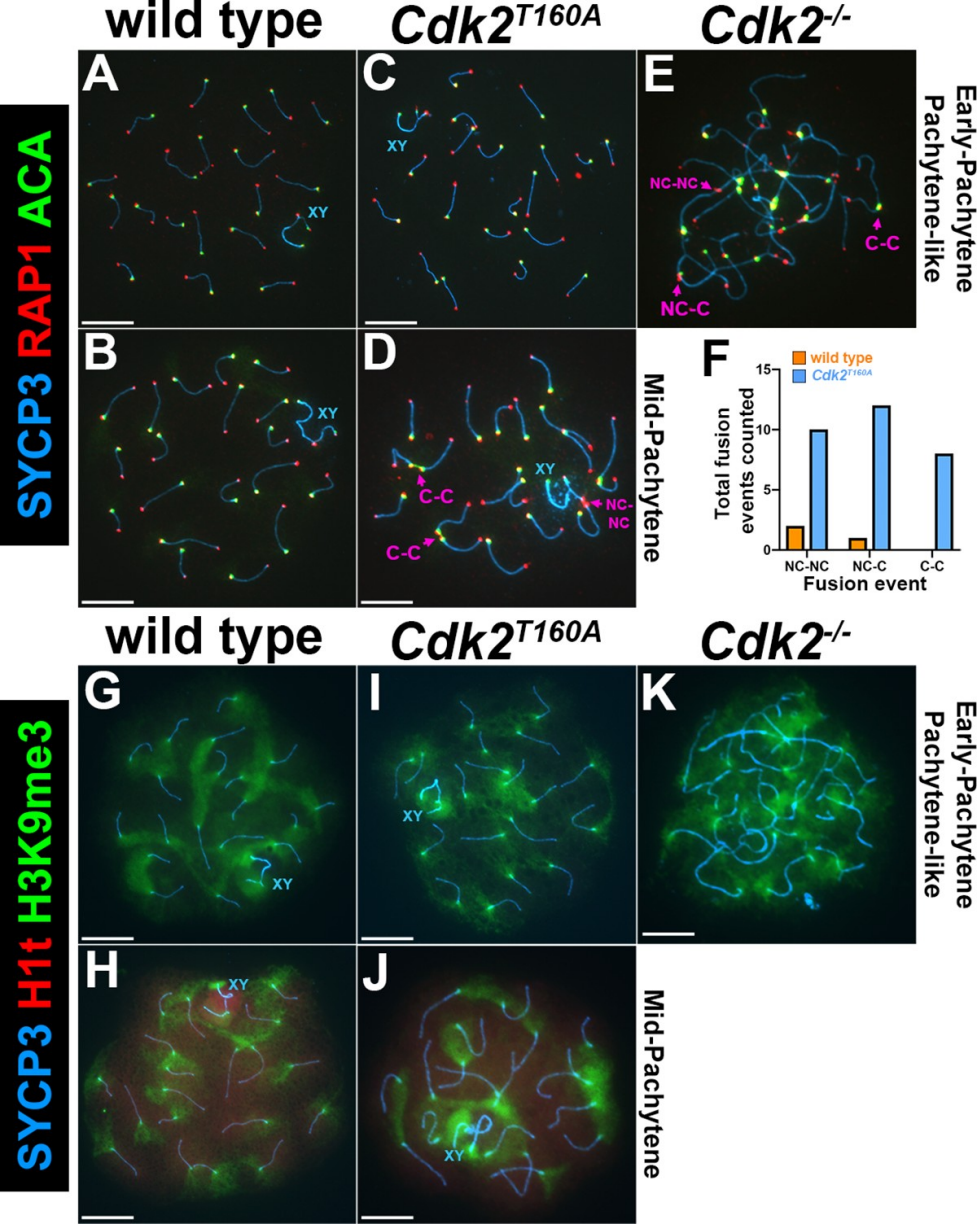
Analysis of telomeric fusion defects in *Cdk2*^*T160A*^ spermatocytes. Chromosome spread preparations from adult (postnatal day 40) testes immunostained with ACA (green) and the telomeric shelterin protein RAP1 (red) in conjunction with SYCP3 (blue) are shown for WT (A–B), *Cdk2*^*T160A*^ (C–D), and *Cdk2*^*−/−*^ (E) for selected stages of meiotic prophase I. For each of the 3 genotypes, RAP1 and ACA can be detected at the telomeres and centromeres as specific foci, respectively. The centromeric ACA signal is always detected proximal to a telomeric RAP1 signal because of the proximity of the centrosome to telomeric ends in mouse chromosomes. In pachytene-like *Cdk2*^*−/−*^ spermatocytes (E) and mid-pachytene–stage *Cdk2*^*T160A*^ spermatocytes (D), telomere fusions can be observed. Quantification of fusion events (F) specifically for mid-pachytene–stage nuclei was performed for WT (orange bars, N = 3 total events counted) and *Cdk2*^*T160A*^ spermatocytes (blue bars, N = 30 total events counted). Here, data are presented as the total number of NC–NC, NC–C, and C–C fusions, respectively. For each genotype, fusion events were counted from 90 images pooled from 3 biological replicates in which at least 20 images were taken from each replicate; no statistical test is applied because of the low numbers of countable events. Examples of C–C, NC–C, and NC–NC fusions are shown in panels D–E by pink arrows. Additional staining is shown for the pericentromeric chromatin marker, H3K9me3 (green) in conjunction with SYCP3 (blue) for WT (G–H), *Cdk2*^*T160A*^ (I–J), and *Cdk2*^*−/−*^ (K), for selected stages of meiotic prophase I. Histone H1t (red) positivity also shown and is indicative of mid-pachytene stage onwards. In all WT and *Cdk2*^*T160A*^ images, pericentric chromatin can be visualized as a flare-like staining emanating from the end of bivalents. In *Cdk2*^*−/−*^, aggregates of H3K9me3-positive pericentromeric chromatin show the extensive interactions between centromeric ends. All images within Fig 2 are representative of at least 20 images taken for specified stages. Similar staining patterns were confirmed in at least 3 biological replicates. In all main panels, scale bars are representative of 5 μm; in all inset pictures, scale bars are representative of 1.25 μm. The underlying data for (F) can be found in S1 Data. ACA, autocentromere antibody; CDK2, cyclin-dependent kinase 2; C–C, centromeric end to centromeric end; H3K9me3, trimethylated lysine 9 of histone H3; NC–C, noncentromeric end to centromeric end; NC–NC, noncentromeric end to noncentromeric end; RAP1, TERF2 interacting protein; SYCP, synaptonemal complex protein; WT, wild-type.

One established role for CDK2 during meiotic prophase is to stabilize interactions between telomeres and the inner nuclear envelope during the homology search process in association with Speedy A. This enables accurate homolog pairing during zygonema [10, 23–25]. The canonical CDK2 binding partners, the E-type cyclins, have also been proposed to maintain telomere stability in meiotic prophase. For example, the deletion of *cyclin E2* has been reported to cause telomeric defects similar to those we observed for the *Cdk2*^*T160A*^ mutant. *Cyclin E2*^−/−^ spermatocytes show both end-to-end associations of bivalents and telomere fusions in both autosomes and sex chromosomes. Although this phenotype is further worsened by the additional deletion of *cyclin E1*, *cyclin E1/2*-deficient spermatocytes are mostly proficient in the completion of synapsis, and the reported meiotic arrest is less severe than can be seen upon deletion of *Cdk2* [13, 48]. To further investigate whether the maintenance of telomere stability might be leading to similar end-to-end defects observed in the *Cdk2*^*T160A*^ mutant, we performed cytological analyses of surface spreads using antibodies against the shelterin complex component RAP1 to detect telomeres. Since centromeres are telocentric in the mouse, we additionally used autocentromere antibodies (ACAs) to distinguish centromeric and noncentromeric ends of chromosomes (Fig 2A–2F). In continuation of our results from Fig 1Q, we found that end-to-end associations between bivalents in mid-pachytene–stage *Cdk2*^*T160A*^ spermatocytes resulted from fusions between telomeres. We categorized fusion events as occurring between telomeres of noncentromeric ends (NC–NC), centromeric ends (C–C), or a mix of both. For clarity, examples of these fusion events are highlighted by pink arrows in Fig 2D and 2E. These fusion events occurred at a similar frequency of 33.3% (NC–NC), 40% (C–C), and 26.6% (mix of NC–NC and C–C), respectively, suggesting that this defect did not predominantly occur at centromeres (Fig 2F). Here, we could not determine the statistical significance because of the relative rarity of mid-pachytene–stage *Cdk2*^*T160A*^ nuclei and the low numbers of these events occurring in WT nuclei.

To further check for potential centromeric defects, we also analyzed the distribution of pericentric heterochromatin using antibodies against trimethylated lysine 9 of histone H3 (H3K9me3) in conjunction with H1t. In pachytene-stage WT spermatocytes, the H3K9me3 signal could be observed as a flare-like pattern emanating from the centromeres of each homolog pair (Fig 2G and 2H). A similar pattern could be observed for pachytene-stage *Cdk2*^*T160A*^ spermatocytes, further suggesting no specific defect in centromeric chromatin for this mutant (Fig 2I and 2J). In contrast, arresting *Cdk2*^*−/−*^ spermatocytes displayed a disordered pattern of H3K9me3 staining, presumably due to the numerous centromeric fusions occurring in these cells (Fig 2K).

### Normal loading of CDK2^T160A^ and Speedy A at telomeres during meiotic prophase I

**Fig 3:**
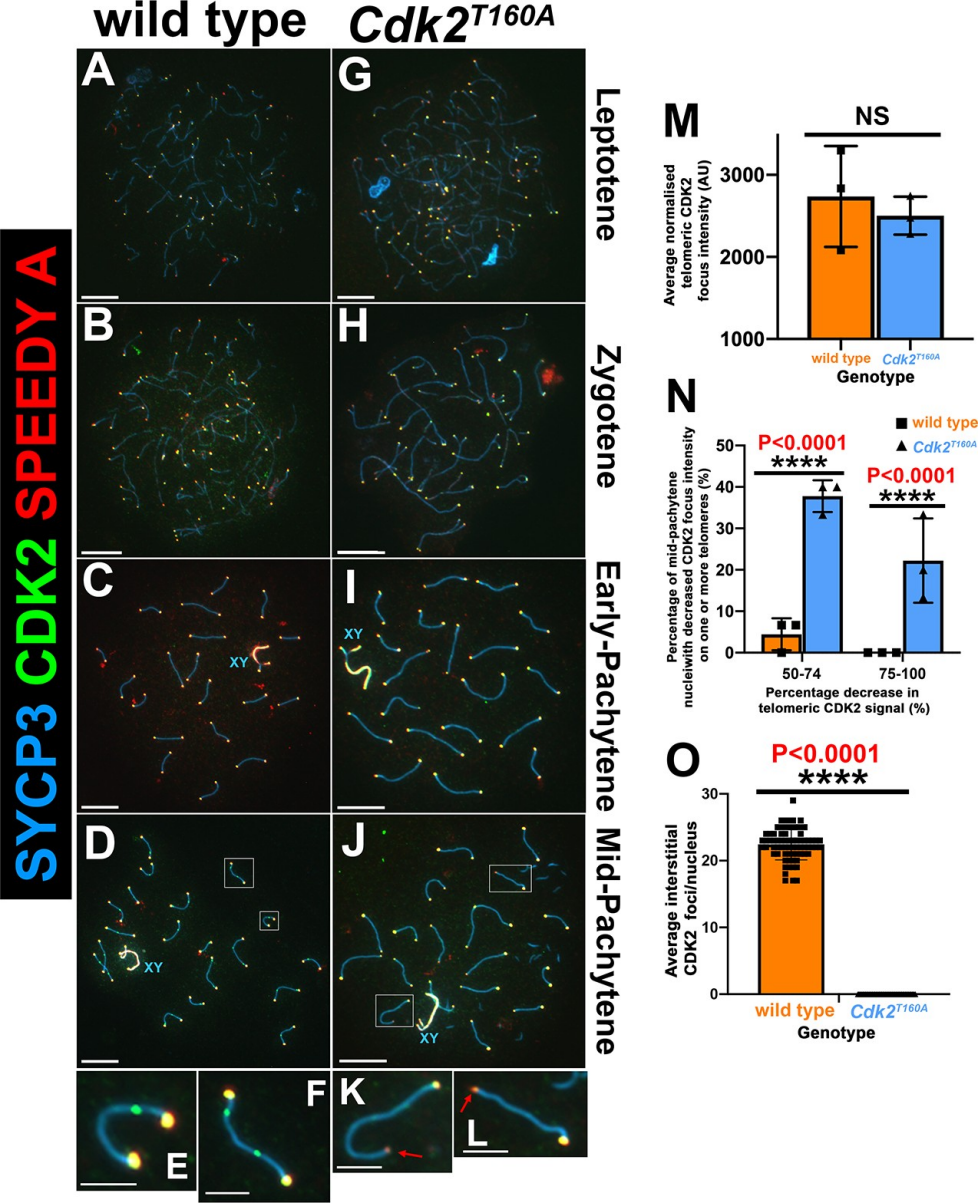
CDK2/Speedy A localization in *Cdk2*^*T160A*^ spermatocytes. Chromosome spread preparations from adult (postnatal day 40) testes immunostained with CDK2 (green) and Speedy A (red) in conjunction with SYCP3 (blue) are shown for WT (A–D) and *Cdk2*^*T160A*^ (G–J) spermatocytes for selected stages of meiotic prophase I. CDK2 and Speedy A can be detected as colocalized foci at all telomeric ends for all meiotic stages in WT (A–D and insets E–F) and *Cdk2*^*T160A*^ spermatocytes (G–J and insets K–L). Interstitial CDK2 foci—absent of Speedy A positivity—marking LRNs are transiently observed in mid-pachytene–stage WT (D) and insets (E–F), but not *Cdk2*^*T160A*^ spermatocytes (J and insets K, L). X–Y bivalents (blue XY labels) were also found to be positive for both CDK2 and Speedy A from early-pachytene onwards in both genotypes. All images are representative of at least 20 images taken for specified stages. Similar staining patterns were confirmed in at least 3 biological replicates. In all main panels, scale bars are representative of 5 μm; in all inset pictures, scale bars are representative of 1.25 μm. Interstitial CDK2 foci (M) were quantified specifically for mid-pachytene stages by counting the average numbers of nontelomeric CDK2 foci counted per nucleus. Data are presented as individual foci counts for WT (orange bars, N = 72) and *Cdk2*^*T160A*^ (blue bars, N = 50); error bars are indicative of the mean and SD. For mid-pachytene, *Cdk2*^*T160A*^ spermatocytes (J) with decreased CDK2/Speedy A signal in a subset of nuclei was observed (as marked by red arrows in insets K, L). The average telomeric CDK2 focus intensity/nuclei were quantified specifically for mid-pachytene stages (N). Data are presented as a mean intensity in AU ± SD determined from 3 biological replicates (N = 48 overall nuclei counted for WT (orange bars) and N = 48 overall nuclei counted (blue bars) for *Cdk2*^*T160A*^. Telomeric signals were only counted from autosomes and were excluded if involved in telomeric fusion events. All intensity values calculated for a single nucleus were normalized to the background intensity of that nuclei. Percentages of mid-pachytene–stage nuclei with at least one telomere showing a decrease in telomeric CDK2 intensity of ≥50% (as compared with the average telomeric CDK2 intensity for that cell) are quantified in panel O. Individual data used to make panel O are shown in S3G Fig. All data were assumed to be non-normally distributed. Statistical significance between genotypes was determined by unpaired *t* test. Significance and *P*-values are reported directly over each comparison. The underlying data for (M, N, O) can be found in S1 Data. AU, arbitrary unit; CDK2, cyclin-dependent kinase 2; LRN, late recombination nodule; SD, standard deviation; SYCP, synaptonemal complex protein; WT, wild-type.

Numerous studies have shown that CDK2 not only localizes to meiotic telomeres but also LRNs [9–16]. Upon deletion of the LRN-associated proteins ring finger protein 212 (*Rnf212*), human enhancer of invasion clone 10 (*Hei10*), proline-rich protein 19 (Prr19; [84]), or cyclin N-terminal domain-containing protein 1 (*Cntd1*), CDK2 localization to recombination nodules is severely or completely depleted. In contrast, telomeric CDK2 binding is unaffected in each of these mutants [11, 16, 49, 84]. Although prior in vitro studies suggested that the CDK2^T160A^ protein can associate with Speedy A to form an active kinase complex [32], this has not been formally tested in vivo. Therefore, we sought to determine whether this mutation would negatively impact the normal localization pattern of CDK2^T160A^ or Speedy A protein during meiotic prophase. In WT and *Cdk2*^*T160A*^ spermatocytes, both Speedy A and CDK2 exhibited normal telomeric localization throughout early meiotic prophase I (Fig 3A–3D and Fig 3G–3J, respectively, and as previously reported for WT [9, 25]). In *Cdk2*^*−/−*^ spreads, Speedy A cannot be observed at telomeric ends, and extensive asynapsis occurs before the observance of a normal pachytene stage (S3A–S3C Fig). This is also true of the converse situation because CDK2 cannot be observed at telomeric ends in *Speedy A*^*−/−*^ spermatocytes, suggesting a codependency for these proteins for the stable binding of telomeric ends (S3D–S3F Fig). This is in agreement with prior findings that Speedy A is required for the loading of CDK2 onto telomeres during early meiotic prophase [24]. Following our prior observations that the mid-pachytene–stage *Cdk2*^*T160A*^ spermatocytes are phenotypically abnormal, we were not able to observe the typical appearance of interstitial CDK2 foci in this model (compare Fig 3D and insets Fig 3E and 3F with Fig 3J and insets Fig 3K and 3L). For WT nuclei, these interstitial foci could be observed at a frequency of 22.4 ± 2.3 (Fig 3O).

Interestingly, we noted that in several mid-pachytene–stage *Cdk2*^*T160A*^ spermatocytes, the intensity of telomeric CDK2 and Speedy A signal was decreased. Globally, the average intensity of telomeric CDK2 signal/nuclei (Fig 3M) or telomeric Speedy A signal/nuclei (S3H Fig) was not significantly different between WT and the *Cdk2*^*T160A*^ mutant. However, when considering the intensity of individual telomeric nuclei (plotted in S3G Fig as a percentage of total signal/nuclei), the *Cdk2*^*T160A*^ mutant clearly displayed a drastic decrease in the intensity of a subset of telomeric nuclei for both CDK2 and Speedy A. Considering only individual telomeric CDK2 signals, 38% ± 4% of *Cdk2*^*T160A*^ mid-pachytene nuclei had at least 1 telomere with a decrease in intensity of 50%–74%, and 22% ± 10% had at least 1 telomere with a decrease of 75%–100% as compared with 4% ± 4% and 0% in WT, respectively (Fig 3N). Similar results were also found in the identical analysis for telomeric Speedy A signals (S3I Fig).

Our above findings indicate that the telomeric localization of the CDK2^T160A^ and Speedy A is broadly unaffected by T-loop mutagenesis of CDK2 during early meiotic prophase. This is an important finding because it suggests that CDK2/Speedy A complexes can act independently of T-loop phosphorylation during meiosis in vivo to promote synapsis. We also uncovered a more specific defect in the *Cdk2*^*T160A*^ mutant in which CDK2 and Speedy A intensities are decreased at a subset of telomeres in mid-pachytene nuclei. The decreased binding of CDK2 and Speedy A at telomeres suggests that the integrity of these regions may be compromised by the T160A mutation. We hypothesize that such a telomeric defect may also in part account for the increased telomeric fusions described for this mutant.

Importantly, we found that interstitial CDK2 foci do not form in the *Cdk2*^*T160A*^ mutant. Although this suggests that the process of LRN formation requires activation of CDK2/complexes via T-loop phosphorylation, we cannot infer from these results whether this was a direct effect, arising from the absent activity of CDK2 at these sites, or indirect, arising from the additional meiotic defects we have described for this model.

### Normal meiotic sex chromosome inactivation (MSCI) and γH2AX dynamics in *Cdk2*^*T160A*^ spermatocytes

**Fig 4:**
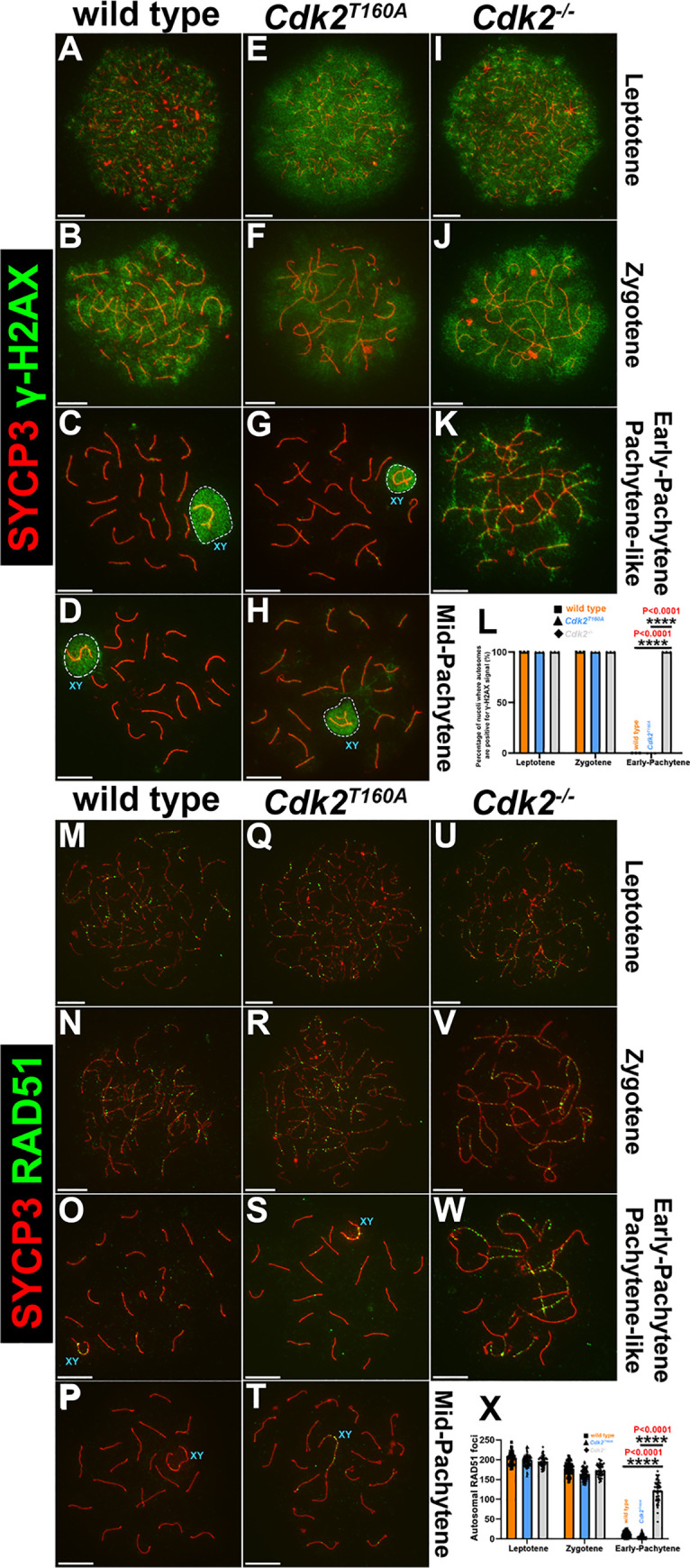
γH2AX analysis of meiotic DSB repair in WT, *Cdk2*^*T160*^, and *Cdk2*^*−/−*^ spermatocytes. Chromosome spread preparations from adult (postnatal day 40) testes immunostained with the DNA damage marker γH2AX (green) and SYCP3 (red) are shown for WT (A–D), *Cdk2*^*T160A*^ (E–H), and *Cdk2*^*−/−*^ (I–K) spermatocytes, for selected stages of meiotic prophase I. γH2AX can be detected as a panchromatin stain in leptotene through zygotene stage in all genotypes and all stages for the *Cdk2*^*−/−*^ mutant. At early-pachytene, γH2AX localizes to the chromatin surrounding the X–Y bivalent (sex body)—outlined with dashed lines—in WT (C) and *Cdk2*^*T160A*^ (G), indicating normal MSCI. This pattern is retained into mid-pachytene (D and H) but does not occur in the *Cdk2*^*−/−*^ mutant (K). Percentages of nuclei retaining γH2AX signal specifically on autosomes, are quantified for all genotypes from leptotene to early-pachytene (L). Data are presented as a mean percentage of cells ± SD determined from 3 biological replicates (N = 60 for the overall nuclei counted for WT leptotene, zygotene, and early-pachytene stages [orange bars]; N = 60 for the overall nuclei counted for *Cdk2*^*T160A*^ leptotene, zygotene, and early-pachytene stages [blue bars]; and N = 60 for the overall nuclei counted for *Cdk2*^*−/−*^ leptotene, zygotene, and early-pachytene stages [gray bars]). Additional staining is shown for P40 chromosome spread preparations immunostained for RAD51 (green) and SYCP3 (red). WT (M–P), *Cdk2*^*T160A*^ spermatocytes (Q–T), and *Cdk2*^*−/−*^ (U–W) images are shown for selected stages of meiotic prophase I. During leptotene and zygotene stages in WT (M–N), *Cdk2*^*T160A*^ (Q–R), and *Cdk2*^*−/−*^ (U–V), RAD51 focus formation precedes the synapsis of homologs visualized using SYCP3. From the early-pachytene stage, fully paired homologs from WT and *Cdk2*^*T160A*^ spermatocytes lose RAD51 foci (O–P and S–T, respectively). In pachytene-like *Cdk2*^*−/−*^ spermatocytes, only a pachytene-like arrest state is achieved (W). Here, RAD51 foci are observed to remain bound to stretches chromosomal axes despite extensive nonhomologous synapsis. These are sites of presumed failed strand invasion events. All images are representative of at least 20 images taken for specified stages. Similar staining patterns were confirmed in at least 3 biological replicates. In all main panels, scale bars are representative of 5 μm. RAD51 foci were quantified specifically for leptotene, zygotene, and early-pachytene stages (X) by counting the average numbers of RAD51 foci per nucleus. Data are presented as individual foci counts for WT (orange bars, N = 90 for leptotene, zygotene, and early-pachytene stages), *Cdk2*^*T160A*^ (blue bars, N = 90 for leptotene, zygotene, and early-pachytene stages) and *Cdk2*^*−/−*^ (gray bars, N = 90 for leptotene, zygotene, and early-pachytene stages). For panels L and X, error bars are indicative of the mean and SD. All data were assumed to be non-normally distributed. Statistical significance between genotypes was determined by unpaired *t* test. Significance and *P*-values are reported directly over each comparison. The underlying data for (L, X) can be found in S1 Data. CDK2, cyclin-dependent kinase 2; DSB, double-strand break; MSCI, meiotic sex chromosome inactivation; RAD51, RAD51 recombinase; SD, standard deviation; SYCP, synaptonemal complex protein; WT, wild-type; γH2AX, phosphoserine 139 H2AX.

As we have established that synapsis progresses in a normal manner in *Cdk2*^*T160A*^ spermatocytes until at least an early-pachytene stage, we considered that a failure to complete repair of DNA intermediates formed after strand invasion might account for the arrest and apoptotic cell death observed in these spermatocytes. A general marker of DSB formation is Ser139 phosphorylation of the histone H2A variant (γH2AX; [50–52]). During meiotic prophase, the γH2AX signal can be observed along unpaired axes (Fig 4A and 4B) and can no longer be detected following the completion of stable strand invasion (Fig 4C and 4D), which occurs in parallel with homolog pairing [53]. By early-pachytene, the γH2AX signal persists only around the XY body (outlined in Fig 4C and 4D), where it functions in the process of MSCI [54, 55]. In *Cdk2*^*T160A*^ spermatocytes, γH2AX exhibited a pattern comparable to WT during meiotic prophase (Fig 4E–4H). By early-pachytene, this signal was correctly restricted to the XY body, and little γH2AX was observed on autosomal chromatin (Fig 4G; compare with WT, Fig 4C). In contrast, γH2AX remains at the chromosome axes even in early-pachytene *Cdk2*^*−/−*^ (Fig 4I–4K). A quantification of γH2AX localization is shown in Fig 4L. Broadly, these results suggest that the initial stages of meiotic DSB repair and MSCI remain unperturbed in *Cdk2*^*T160A*^ spermatocytes and are supportive that early events of meiotic prophase are not overtly affected by this point mutation.

### Normal strand invasion in *Cdk2*^*T160A*^ spermatocytes

During meiotic prophase, successful interhomolog recombination requires the formation of homotypic nucleofilaments formed of RAD51 recombinase (RAD51) and dosage suppressor of Mck1 homolog (DMC1) recombinase at single-stranded DNA exposed by the resection of meiotic DSBs [56]. Here, RAD51/DMC1 nucleofilaments facilitate the strand invasion of a nonsister chromatid on a homologous chromosome. RAD51/DMC1 nucleofilament formation occurs before the completion of synapsis, and numerous foci of RAD51/DMC1 can be observed on unpaired axes in leptotene and early zygotene stages (Fig 4M and 4N). Following synapsis in early-pachytene (Fig 4O), successful strand invasion is thought to allow RAD51/DMC1 dissociation from chromosomal axes (see [57–60]). To determine whether this process occurs as normal in *Cdk2*^*T160A*^ spermatocytes, we stained for RAD51 and SYCP3. We observed that the dynamics of RAD51 foci were largely similar between WT and *Cdk2*^*T160A*^ spermatocytes. Numerous RAD51 molecules were observed to bind to unpaired axes in zygotene and are subsequently lost from paired axes. By early-pachytene, when homologs are fully paired, RAD51 foci were no longer detectable (*Cdk2*^*T160A*^, Fig 4Q–4S; compare with WT, Fig 4M–4O).

In *Cdk2*^*−/−*^ spermatocytes, it has previously been established that RAD51 foci persist into a pachytene-like state, suggesting that strand invasion is incomplete in this model ([10], Fig 4U–4W, and S4K Fig). The normal disappearance of RAD51 foci during the zygotene-pachytene transition in *Cdk2*^*T160A*^ spermatocytes (Fig 4R and 4S) is indicative of normal recombination intermediate formation and resolution occurring in parallel with successful homolog synapsis. Quantification of the data is shown in Fig 4X.

For a more in-depth analysis of the progression of meiotic recombination in the *Cdk2*^*T160A*^ mutant, we performed further analysis of the localization of the replication protein A (RPA2). This protein comprises the 32-kDa subunit of the trimeric replication protein A complex (RPA). Following RAD51/DMC1 dissociation, RPA remains localized to early sites of recombination. RPA2 foci decreased in number during the progression of prophase I and were eventually not discernible in late-pachytene of WT spermatocytes (S4A–S4D Fig). The progressive decline in RPA foci is thought to coincide with the repair of early recombination nodules as NCO events [61–63]. No significant differences in RPA2 focus counts could be found between WT, *Cdk2*^*T160A*^, and *Cdk2*^*−/−*^ spermatocytes in the leptotene, zygotene, or early-pachytene stages (S4E–S4K Fig). However, during mid-pachytene, a significant decrease in RPA2 foci was observed in WT spermatocytes, whereas RPA2 focus numbers remained high in *Cdk2*^*T160A*^ spermatocytes (71 ± 27 versus 197 ± 11, *P* < 0.0001) (S4L Fig). The patterning of RPA staining in *Cdk2*^*T160A*^ spermatocytes was comparable with that observed for *Cdk2*^*−/−*^ spermatocytes in a pachytene-like arrest state, and established foci appeared to persist stably (compare to S4G and S4K Fig). Here, the stable/persistent nature of RPA foci in both the *Cdk2*^*T160A*^ and *Cdk2*^*−/−*^ models suggests that these cells lack specific signals or cellular events that are required to trigger the repair process at stabilized strand intermediates.

### Persistence of MSH4 and RNF212 foci in *Cdk2*^*T160A*^ spermatocytes

Following strand invasion, early recombination intermediates are thought to be stabilized by the MutSγ DNA clamp complex composed of MutS homolog proteins 4 and 5 (MSH4/MSH5) [46, 47, 64]. This stabilizing event is required both for the completion of homolog synapsis and also the downstream formation of meiotic COs. During this process, RAD51/DMC1 foci are typically replaced by MSH4 foci upon normal synapsis [7, 46, 65].

**Fig 5:**
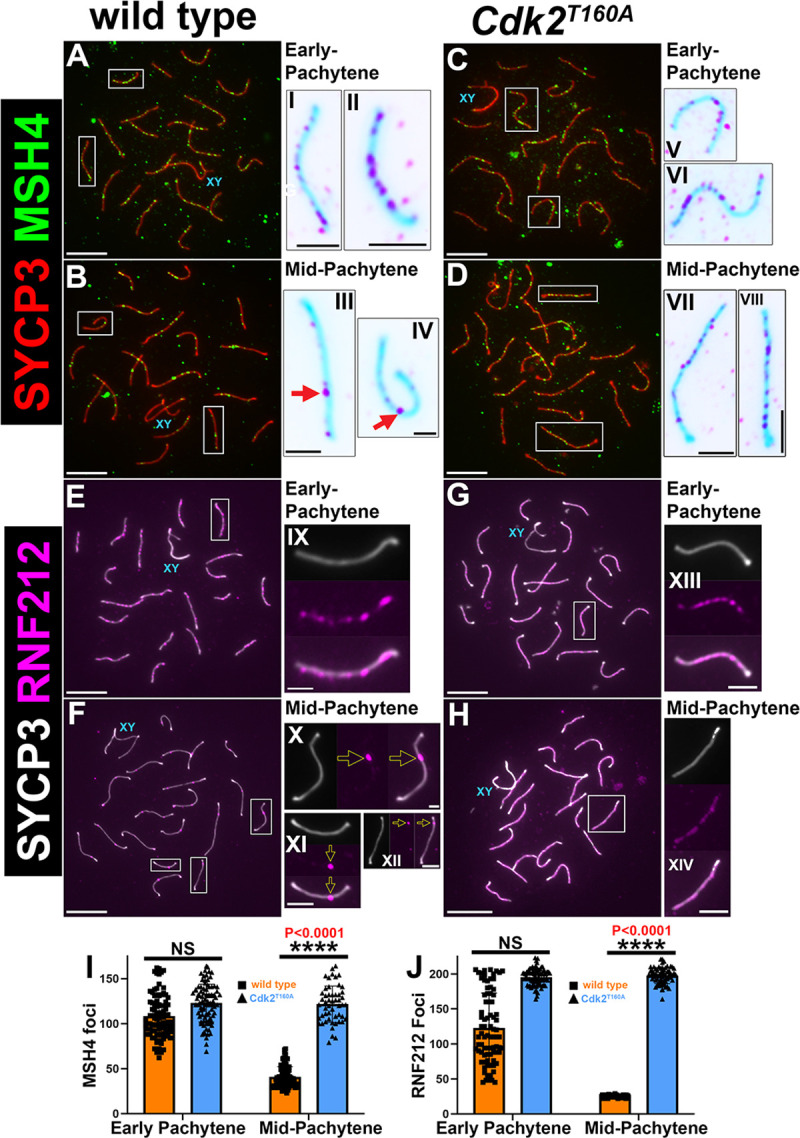
Comparison of MSH4 and RNF212 dynamics in WT and *Cdk2*^*T160A*^ spermatocytes. P40 chromosome spread preparations immunostained for MSH4 (green) and SYCP3 (red) are shown for WT (A–B) and *Cdk2*^*T160A*^ spermatocytes (C–D) for selected stages of meiotic prophase I. Inset images are shown in their corresponding inverted color for better visualization of MSH4 foci. In early-pachytene WT and *Cdk2*^*T160A*^ spermatocytes, MSH4 foci can be observed along the length of paired axes (A and C, insets I–II and V–VI). MSH4 foci decrease in number as WT spermatocytes progress to mid-pachytene (B, insets III–IV). MSH4 focus numbers remain high in mid-pachytene *Cdk2*^*T160A*^ spermatocytes (D, insets VII–VIII). An identical analysis is shown for P40 chromosome spread preparations immunostained for RNF212 (magenta) and SYCP3 (white). Like MSH4, RNF212 foci in early-pachytene WT and *Cdk2*^*T160A*^ spermatocytes can be observed along the length of paired axes (E and G, insets IX and XIII). RNF212 foci decrease in number as WT spermatocytes progress to mid-pachytene. At this stage, RNF212 foci mark LRNs (F, insets X–XII). RNF212 focus numbers remain high in mid-pachytene *Cdk2*^*T160A*^ spermatocytes (H, inset XIV). All images are representative of at least 20 images taken for equivalent stages. Similar staining patterns were confirmed in at least 3 biological replicates. In all main panels, scale bars are representative of 5 μm; in all inset pictures, scale bars are representative of 1.25 μm. Quantification of MSH4 foci and RNF212 foci counts are shown in panels I and J, respectively. Data are presented as individual foci counts, and WT and *Cdk2*^*T160A*^ data are represented using orange or blue bars, respectively. Error bars are indicative of the mean and SD. All data were assumed to be non-normally distributed. Statistical significance between genotypes was determined by unpaired *t* test. Significance and *P*-values are reported directly over each comparison. For WT data in I and J, N = 90 for both early-pachytene and mid-pachytene stages. For *Cdk2*^*T160A*^ data in I, N = 90 for early-pachytene and N = 42 for mid-pachytene stages. For *Cdk2*^*T160A*^ data in J, N = 90 for early-pachytene and N = 45 for mid-pachytene stages. The underlying data for (I, J) can be found in S1 Data. CDK2, cyclin-dependent kinase 2; LRN, late recombination nodule; MSH4/5, MutS protein homolog 4/5; RNF212, ring finger protein 212; SD, standard deviation; SYCP, synaptonemal complex protein; WT, wild-type.

To determine the status of MutSγ localization, we stained *Cdk2*^*T160A*^ spermatocytes for MSH4. MSH4 foci were observed specifically at synapsed portions of the SC in a manner indistinguishable from WT (compare Fig 5A and insets I–II to 5C and insets V–VI). Quantification of MSH4 focus numbers in early pachynema indicated that *Cdk2*^*T160A*^ spermatocytes had slightly greater numbers of detectable SC-associated MSH4 foci (123 ± 21) as compared to WT (108 ± 1). By mid-pachytene, this was significantly decreased to 41 ± 11 in WT but remained essentially unchanged in *Cdk2*^*T160A*^ spermatocytes (121 ± 20) (see Fig 5I and compare panels 5B and 5D). By mid-pachytene, it is established that the majority of MSH4 foci colocalize with MutLγ at incipient CO sites [7]. By late-pachytene, no MSH4 foci could be observed in WT. Our data support prior studies that suggest MSH4 colocalizes with a subset of early recombination nodules (identified in this study using RPA) [61,66].

A prime candidate responsible for the persistent stabilization of RPA/MSH4 marked recombination intermediates in *Cdk2*^*T160A*^ spermatocytes is the E3 smal ubiquitin-like modifier (SUMO) ligase RNF212. During meiotic prophase I, RNF212 is thought to stabilize early DNA recombination intermediates formed following strand invasion [11]. At the subset of recombination sites that RNF212 colocalizes with MutSγ, this protein has been implicated in directing sites of recombination away from repair as NCOs in favor of biased class I CO formation. In support of our observations that early recombination intermediates persist in *Cdk2*^*T160A*^ spermatocytes, we observed that RNF212 was detected as persistent foci decorating fully paired chromosomal axes in early-pachytene (Fig 5G and inset XIII). This pattern was fairly similar to that seen for WT spermatocytes of the same stage (compare with Fig 5E and inset IX). At this point, although the number of MSH4 foci counted in WT was lower than that seen in *Cdk2*^*T160A*^ spermatocytes, this was not significant because of the variability seen in WT focus numbers (126 ± 50 as compared with 196 ± 12). As meiotic prophase progresses from early to mid-pachytene, RNF212 foci decrease drastically until only a subset of 25 ± 2 remains in WT nuclei (Fig 5F and marked by yellow arrows in insets X–XII). These represent the LRNs that will eventually form meiotic COs and are essentially identical to the frequencies of CDK2 reported for WT mid-pachytene spermatocytes in Fig 3O. Such a decrease in RNF212 focus number was never observed in mid-pachytene *Cdk2*^*T160A*^ spermatocytes, which remained at the frequency of 198 ± 11 (compare Fig 5H–5J with 5F). In contrast with WT RNF212 foci of roughly uniform intensity and size, none of the initial RNF212 foci seen in early-pachytene reach a “mature” state in *Cdk2*^*T160A*^ spermatocytes. This maturation is likely dependent upon the normal activity of CDK2 in the mid-pachytene state, which is compromised by the T-loop mutagenesis of CDK2.

### MutLγ foci are not formed in *Cdk2*^*T160A*^ spermatocytes

Our results showing that the early-recombination-nodule–associated proteins, including RPA2, MSH4, and RNF212, persist at high numbers along paired chromosomal axes, in addition to the failure of CDK2 localization to LRNs, were indicative that the CO formation process was impaired in the *Cdk2*^*T160A*^ model.

**Fig 6:**
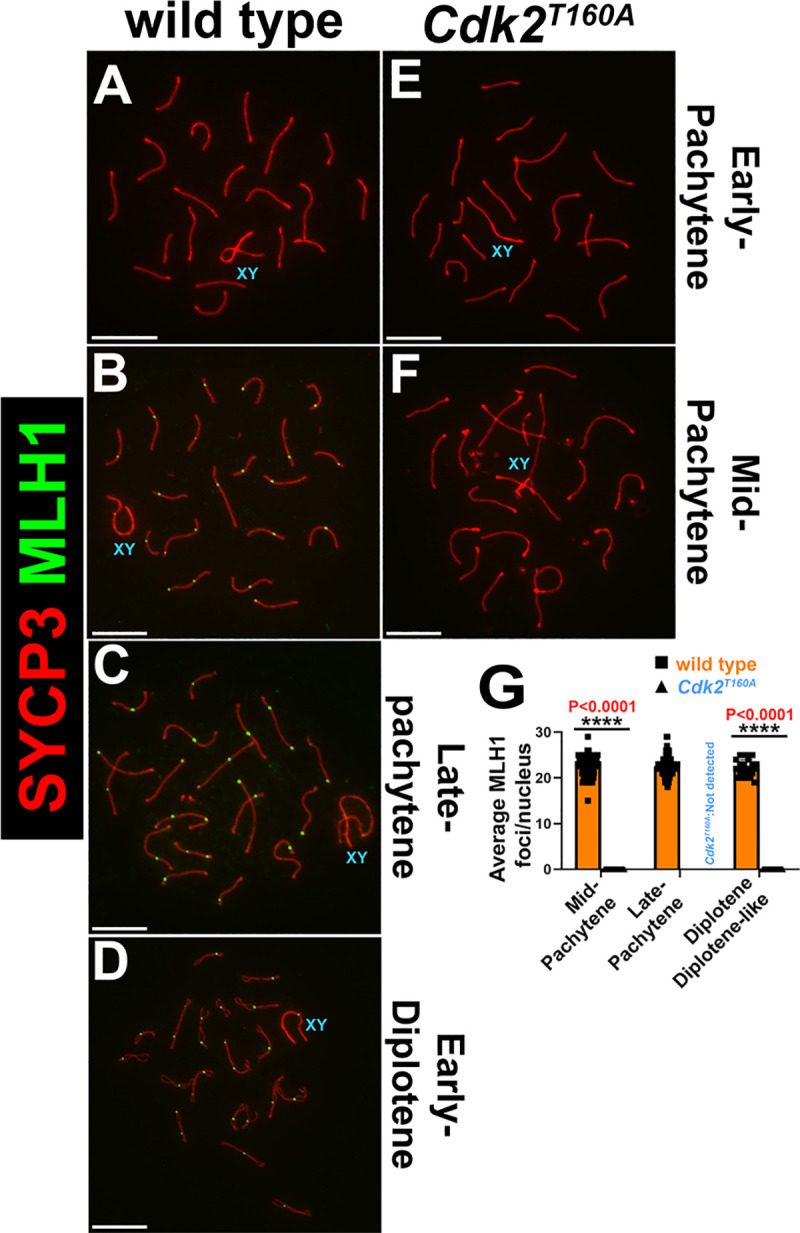
Analysis of MLH1 dynamics in *Cdk2*^*T160A*^ spermatocytes. Chromosome spread preparations from adult (postnatal day 40) testes immunostained for MLH1 (green) and SYCP3 (red) are shown for WT (A–D) and *Cdk2*^*T160A*^ (E–F) for selected stages of meiotic prophase I. Positivity for MLH1 is seen in mid-pachytene through diplotene in WT (C, D) but could not be detected in any *Cdk2*^*T160A*^ stage. Images are representative of at least 20 images taken for equivalent stages. Identical staining patterns were confirmed in at least 3 biological replicates. Scale bars are representative of 5 μm. MLH1 foci were quantified specifically for mid-pachytene, late-pachytene, and diplotene/diplotene-like stages (G) by counting the average numbers of MLH1 foci per nucleus. Data are presented as individual foci counts for WT (orange bars, N = 90 for mid-pachytene, late-pachytene, and diplotene stages) and *Cdk2*^*T160A*^ (blue bars, N = 72 for mid-pachytene and N = 60 for diplotene-like stages; late-pachytene stages were not detected in this genotype). Error bars are indicative of the mean and SD. All data were assumed to be non-normally distributed. Statistical significance between genotypes was determined by unpaired *t* test. Significance and *P*-values are reported directly over each comparison. The underlying data for (G) can be found in S1 Data. CDK2, cyclin-dependent kinase 2; MLH1, MutL homolog 1; SD, standard deviation; SYCP, synaptonemal complex protein; WT, wild-type.

To definitively determine the effect on CO formation in *Cdk2*^*T160A*^ spermatocytes, we stained for the cytological marker of class I interference-dependent COs, MutL homolog 1 (MLH1), in conjunction with SYCP3 [67, 68]. In WT spermatocytes, MLH1 foci cannot be observed until mid-pachytene, when they were observed at a frequency of 1–2 per autosome (Fig 6A–6G). A singular MLH1 signal was also evident within the PAR of the X–Y chromosomes, totaling an average of 22.5 ± 2.1 MLH1 foci per mid-pachytene nucleus (in all analyzed nuclei, no chromosome pairs were observed with 3 or more MLH1 foci, which is consistent with previous studies [69–71]). MLH1 foci persisted until diplonema in WT (22.5 ± 2.2 in late-pachytene and 22.2 ± 1.8 in diplotene; Fig 6G). In contrast, no MLH1 foci could be observed at any meiotic stage present in *Cdk2*^*T160A*^ spermatocytes (an example of a mid-pachytene–stage *Cdk2*^*T160A*^ nuclei is shown in Fig 6F for reference). One possible reason for this could be a failure to resolve early recombination intermediates to form COs or NCOs, preventing the normal completion of meiotic prophase. This result highlights the limitations of our “partial-loss-of-function” mutant, and therefore, other approaches are necessary to continue these investigations. We sought to further investigate this by using a “gain-of-function” mutation in CDK2 (see below).

### CDK2 hyperactivity results in increased accumulation of CDK2 and MLH1 foci at interstitial sites

By utilizing the partial-loss-of-function *Cdk2*^*T160A*^ model, we can draw several conclusions. Firstly, the activating phosphorylation of CDK2 on Thr160 is not required for homolog synapsis. Similarly, this mutation does not preclude normal strand invasion mediated by RAD51/DMC1 at meiotic DSBs. Secondly, activating phosphorylation of CDK2 on Thr160 is essential for the repair of recombination intermediates formed at meiotic DSBs. This second point suggests that CDK2 activity might be required for the conversion of recombination intermediates to form meiotic COs. However, we could not conclusively show this because of the failed designation of early recombination intermediates, which typically precedes the formation of LRNs.

To overcome this restriction, we took advantage of the *Cdk2*^*Y15S*^ mice, which we have previously shown to display increased CDK2 kinase activity [17]. Although adult *Cdk2*^*Y15S*^ animals are devoid of spermatocytes because of a loss of stem cell renewal, spermatocytes can be seen to progress through meiotic prophase I during the first wave of spermatogenesis in juvenile animals. During the first wave of spermatogenesis, *Cdk2*^*Y15S*^ spermatocytes achieve extensive homologous chromosome synapsis. This is shown by SYCP3/SYCP1 colocalization in S5B,S5E and S5H Fig in comparison with WT (S5A,S5D and S5G Fig). In particular, >85% of pachytene-stage nuclei show a full complement of synapsed homologs (S5J Fig). However, mid- and late-pachytene–like *Cdk2*^*Y15S*^ spermatocytes exhibited a mild nonhomologous synapsis phenotype in which some bivalents appeared fused at ends (85.3 ± 5 in Y15S compared to 0.9 ± 1.4 in WT spermatocytes; S5K Fig). Despite γH2AX positivity, the sex body of *Cdk2*^*Y15S*^ spermatocytes did not seem to achieve the level of compaction observed in WT spermatocytes, as evidenced by the diffuse γH2AX signal associated with these structures (as highlighted by the dashed line in S5E Fig). Similar compaction defects have recently been noted in point mutants of γH2AX (*H2ax*^*Y142A*^), which are defective in normal MSCI [72]. Similar to the T160A mutation, the Y15S mutation was permissive of grossly normal strand invasion, as RAD51 foci were observed to be depleted from chromosomal axes upon completion of synapsis (S5H Fig). As seen in the *Cdk2*^*T160A*^ mutant, in *Cdk2*^*Y15S*^ spermatocytes, the RAD51 focus numbers were not significantly different from WT following their synapsis (S5L Fig). In contrast to singular Y15S or T160A mutation, compound mutation of Y15 and T160A of *Cdk2* (Y15S/T160A) resulted in a meiotic arrest phenotype (S5C, S5F and S5I Fig) indistinguishable from that already reported for *Cdk2*^*−/−*^ animals [10, 27, 28].

**Fig 7:**
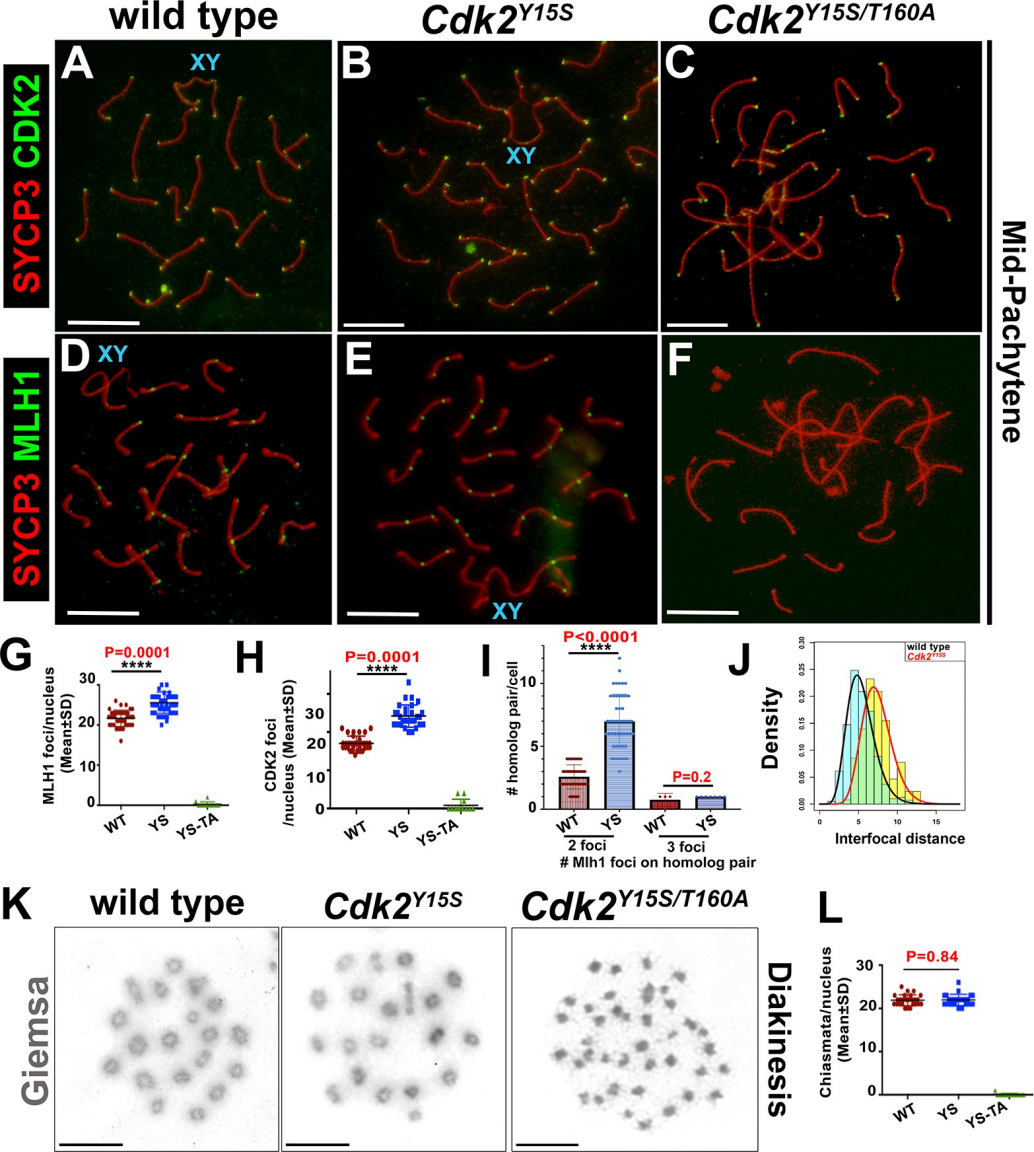
Analysis of MLH1 dynamics in *Cdk2*^*Y15S*^ and *Cdk2*^*Y15S/T160A*^ spermatocytes. Chromosome spread preparations from pubertal (postnatal day 25) testes immunostained for CDK2 or MLH1 (green) and SYCP3 (red) are shown for WT (A,D), *Cdk2*^*Y15S*^ (B,E), and *Cdk2*^*Y15S/T160A*^ (C,F) pachytene spermatocytes. Interstitial CDK2 and MLH1 foci can be detected in *Cdk2*^*Y15S*^, but not *Cdk2*^*Y15S/T160A*^, spermatocytes, which do not reach the equivalent meiotic stage. Images are representative of at least 20 nuclei imaged at equivalent stages. Identical staining patterns were confirmed in at least 3 biological replicates. Scale bars = 5 μm. Quantification of MLH1 foci, CDK2 foci, and frequency of homologs with 2 or 3 foci in indicated genotypes are shown in panels G, H, and I, respectively. Gamma distribution analysis of MLH1 foci along bivalents (J). Giemsa staining of diakinesis preparations from WT and *Cdk2*^*Y15S/Y15S*^ gonads showing comparable chiasmata with 20 bivalent chromosomes in either genotype (K). Chiasmata numbers for each cell as well as average counts (+SD) for WT and Y15S are shown in (L). Data from age-matched WT animals are shown for comparison. Individual points are pooled from at least 3 biological replicates; error bars are representative of SD. Interfocus distances between MLH1 foci on (chromosomes with >1 MLH1 focus) are quantified for each genotype in panel J for WT and *Cdk2*^*Y15S*^ spermatocytes. The underlying data for (G, H, I, J, L) can be found in S1 Data. CDK2, cyclin-dependent kinase 2; MLH1, MutL homolog 1; SD, standard deviation; SYCP, synaptonemal complex protein; WT, wild-type; YS, *CDK2*^*Y15S*^ genotype; YS-TA, *CDK2*^*Y15S/T160A*^ genotype.

To explore whether elevation of CDK2 kinase activity [17] was permissive of crossing over, we performed immunocytological analyses of CDK2 and MLH1 foci on surface-spread spermatocyte nuclei from juvenile (P25) mice (Fig 7A–7J). In contrast to *Cdk2*^*−/−*^ and *Cdk2*^*T160A*^ spermatocytes, *Cdk2*^*Y15S*^ spermatocytes progressed to cytologically “normal” mid-/late-pachytene–like stages, as evidenced by the observation of interstitial CDK2 and MLH1 foci in addition to H1t positivity (Fig 7B–7E). Strikingly, *Cdk2*^*Y15S*^ late-pachytene–like spermatocytes had significantly more MLH1 foci than WT (26.1 ± standard deviation [SD] 2.1 versus 21.5 ± SD 2.1, respectively; *P* < 0.0001). This equates to an average increase of 4.6 foci per late-pachytene spermatocyte (quantified in Fig 7G). Similarly, we also observed an approximately 1.4-fold increase in interstitial (nontelomeric) CDK2 foci in *CDK2*^*Y15S*^ spermatocytes (17.1 ± SD 1.9 in WT versus 24.2 ± SD 1.4 in *CDK2*^*Y15S*^) (quantified in Fig 7H). This suggests that CDK2 localization to meiotic recombination intermediates increases with its kinase activity and may be a major factor in their eventual repair as meiotic COs as opposed to NCOs.

As found for *Cdk2*^*T160A*^ spermatocytes, the CDK2 protein could be detected on the telomeres of *Cdk2*^*Y15S/T160A*^ spermatocytes, but not at interstitial sites marking LRNs (Fig 7C). This was also accompanied by the failure to form MLH1 foci (Fig 7F). Because the *Cdk2*^*Y15S/T160A*^ spermatocytes do not reach a normal mid-pachytene stage, we cannot currently conclude whether the T160A mutation is the direct cause of the failure of CDK2 to localize to LRNs, but it is one of the possibilities that can be considered.

The number of class I CO events are influenced by changes in the length of the SC axis. A positive relationship between SC length and CO number has been described for several organisms [73–77], including humans [78] and mice [79]. Given the normal synapsis of mutant chromosome SC axes, we further tested whether the elevated numbers of putative class I COs could be subject to interference. We measured interfocus distances of MLH1 foci and fitted data to a gamma distribution [80]. The distance between MLH1 foci in chromosomes with 2 or more COs in spermatocytes from *Cdk2*^*Y15S*^ and WT was 5.04 ± SD 0.71 versus 7.05 ± SD 1.28, respectively (*P* < 1.07 × 10^−9^) (Fig 7J). Even considering the minor decrease in total chromosome length (11.53 ± SD 1.20 for WT versus 10.07 ± SD 1.14 for Y15S, respectively; *P* < 2.98 × 10^−6^), these data suggest that the distance between 2 MLH1 foci is significantly decreased in the Y15S mutants. In addition, we observed an increased frequency of 2 MLH1 foci on a homolog pair (Fig 7I), but not of 3 MLH1 foci. Because of the increased MLH1–CDK2 focus frequency in *Cdk2*^*Y15S*^ spermatocytes and given the role of these proteins in marking ultimate sites of the LRNs representing CO in other higher eukaryotes, we further examined occurrences of “true” CO events by chiasmata analysis on diakinesis spread preparations. Surprisingly, there was no significant difference in the number of chiasmata in WT and *Cdk2*^*Y15S*^ diakinesis spreads (*P*   =  0.84; Fig 7K and 7L), indicating normal rates of CO in the absence of phosphorylation of Tyr15 on CDK2. Collectively, these data suggest that CDK2 activity is playing a role in proper meiotic COs.

Our results indicate that increased CDK2 activity is correlated with elevated numbers of LRN-associated CDK2 and MLH1 foci in a manner that is associated with decreased MLH1 interfocus distancing. Despite this result, the inability for us to detect increased chiasmata numbers in the *Cdk2*^*Y15S*^ mutant indicates that these apparent LRNs may be nonfunctional and may not be converted into meiotic COs (as we detected via the chiasmata counts in diakinesis spreads), despite the observation of increased CDK2 and MLH1 foci. This is surprising because MLH1 focus counts are thought to accurately mirror the total numbers of class I meiotic CO sites per nuclei [81–83].

In the converse situation, whereby CDK2/cyclin activity is abrogated by T-loop mutagenesis (in the case of both T160A and compound Y15S/T160A mutation), meiosis does not progress to a typical mid-pachytene stage when MLH1 typically localizes to incipient CO sites. Although this could similarly indicate that CDK2 is required for the selection of meiotic CO sites, at this point we cannot rule out the possibility that defects in meiotic progression independent of the CO formation process might arrest meiotic progression in a manner that precludes normal MLH1 foci formation.

## Discussion

Our study suggests that CDK2 activity is an important factor mediating firstly, the maintenance of synapsis; secondly, telomere stability during meiotic prophase; and finally, the normal execution of meiotic recombination to generate COs from early recombination intermediates. Specifically, we find that unlike complete *Cdk2* deletion, T-loop mutagenesis of CDK2 at Thr160 is permissive of both homolog synapsis and MSCI during early meiotic prophase. The normal synapsis observed in this model is likely enabled by localization of both CDK2^T160A^ and its noncanonical binding partner Speedy A to the telomeric ends during early meiotic prophase. Additionally, these results support the idea that CDK2/Speedy A can form a biologically functional complex in vivo in the absence of Thr160 phosphorylation, an idea that to date only had been tested in vitro [34]. The observance of specific defects in both the SC scaffold and the telomeres of paired homologs in mid-pachytene, however, suggests that additional CDK2 complexes beyond CDK2/Speedy A are required for the maintenance of these structures. Indeed, T-loop mutagenesis of CDK2 was associated with decreased binding of CDK2 and Speedy A at telomeres during this stage in selected chromosomes. This coincided with the observance of telomere fusion events, suggesting that the normal environment of the telomeres could be altered by this point mutation. Interestingly, decreased telomeric stability, premature SC desynapsis, and telomere fusions such as seen in the *Cdk2*^*T160A*^ mutant are shared with meiosis-specific *cyclin E* knockout models [13, 48]. Because the E-type cyclins are known binding partners of CDK2, it could be possible that CDK2/cyclin E activity is required to maintain the stability of the SC and telomeres beyond early-pachytene. Although the observed synaptic defects are possible explanations for the meiotic arrest and apoptotic cell death seen in *Cdk2*^*T160A*^ spermatocytes, another possible explanation is a regulatory arrest arising from the failed repair of meiotic recombination intermediates in this model. Aside from the synaptic defects of the *Cdk2*^*T160A*^ mutant, we also found that the normal repair of recombination intermediates, formed following strand invasion at meiotic DSB sites, is incomplete. The typical reduction in early-recombination-nodule–associated proteins, including RPA2, RNF212, and MSH4, that is associated with the “selection” of meiotic CO sites did not occur in the *Cdk2*^*T160A*^ mutant. The persistence of these DNA-damage–associated proteins may trigger cellular checkpoints to remove the afflicted cells. In either case, this arrest of meiotic progression ultimately leads to the apoptotic loss of the majority of pachytene-stage spermatocytes, causing a resultant block in spermatogenesis and male infertility. Finally, our data suggest that CDK2 activity is required to regulate the number of MLH1 foci at meiotic COs forming from LRNs. Specifically, *Cdk2*^*Y15S*^ mutant spermatocytes with increased CDK2-associated activity were found to have greater overall numbers of MLH1 foci. This provocative result is suggestive that CDK2 might phosphorylate specific meiotic substrates that dictate the repair process of recombination intermediates, directing them towards CO formation. Several of the open questions raised by this study are detailed below.

### Does CDK2 promote CO formation?

One of the primary aims of this study was to determine the role of CDK2 at LRNs. A major finding that we believe sheds light on this question was the increased numbers of interstitial CDK2 foci observed in mid-pachytene–stage *Cdk2*^*Y15S*^ spermatocytes, indicating that CDK2 kinase activity is one requirement for the binding of CDK2 to interstitial sites. Prior to this study, it has been shown that the localization of CDK2 to LRNs is defective in many mouse models lacking the so-called “pro-CO” factors RNF212, HEI10, proline-rich protein 19 (PRR19), and CNTD1, which colocalize with late-recombination–associated CDK2 foci under normal conditions. In knockout models for each of these proteins, telomeric CDK2 binding is unaffected, and homolog synapsis is observed to proceed as normal [11, 16, 49]. Recent evidence suggests that CDK2 may be able to interact with the pro-CO factors CNTD1, a cyclin-like protein, and another putative pro-CO factor PRR19—a proline-rich protein of unknown function—to form specific complexes during the meiotic prophase [84]. The cyclin subunit of the CDK/cyclin complex is an important determinant of substrate specificity [85–88]. In situations in which CDKs are complexed with noncanonical activators such as Speedy A, a greater range of substrates can be targeted by the CDK because of the less strict consensus sequence required for phosphorylation by such complexes [34, 89, 90]. It is tempting to speculate that meiosis-specific CDK2 complexes, perhaps in association with CNTD1 and/or PRR19 or other activating proteins, might allow for the targeting of specific substrates at LRNs to mediate CO determination. The relative importance of these proteins for crossing over is not yet reported and thus requires further study to determine their relationship with CDK2.

A potential CDK2 substrate is the E3-ubiquitin ligase HEI10. Although HEI10 lacks a conventional CDK consensus site for phosphorylation, this protein has been suggested as a CDK substrate in a prior publication [16]. It is hypothesized that the SUMOylation of meiotic proteins at early recombination nodules through the SUMO ligase activity of RNF212 mediates their stability. In contrast, ubiquitination by HEI10 is thought to mediate their degradation. The function of HEI10 during zygonema limits the colocalization of RNF212 with MutSγ-associated recombination sites and thereby establishes early differentiation of CO and NCO sites [11, 14]. Later, HEI10 may be directed by CDK2 to stably accumulate at designated CO sites. Upon deletion of *Hei10*, RNF212 foci remain at high numbers despite normal synapsis of homologs, a feature also shared with the *Cdk2*^*T160A*^ mutant [14]. Therefore, it is possible that CDK2 and HEI10 work either within the same pathway or are dependent upon each other to promote the selection of RNF212-marked sites that will become LRNs.

### Implications of increased MLH1 foci observed in the *Cdk2*^*Y15S*^ model

We previously reported that in *Cdk2*^*Y15S*^ mice, the first prepubertal round of spermatogenesis occurs and progresses into meiotic prophase I. Subsequent waves of spermatogenesis in *Cdk2*^*Y15S*^ animals do not progress to meiotic prophase because of a defect in spermatogonial stem cell differentiation [17]. The first round of spermatogenesis originates from prospermatogonia cell types. This is in contrast with adult spermatogenesis, which is initiated by the differentiation of A-type spermatogonia [91]. Thus, the differentiation of A-type spermatogonia, but not prospermatogonia, is perturbed by elevated CDK2 kinase activity caused by *Cdk2*^*Y15S*^ mutation. One of the more provocative observations made in our current study was the increase in MLH1 foci observed in the “hyperactive” *Cdk2*^*Y15S*^ model. Very few instances have been described in which MLH1 foci are observed outside the expected range of 1–2 foci per autosome. Interestingly, a common theme to such meiotic arrest models is the loss of proteins thought to be involved in the formation of “back-up” class II COs that are not subject to interference. One such example is mice lacking the CO junction endonuclease MUS81 (MUS81). Although the majority of COs are dependent upon the localization of MLH1 and MLH3 [67, 68], a small number (5%–10%) of COs can be observed even in mice lacking these proteins [92–94]. These residual COs, which are interference independent, are thought to be mediated by the MUS81/essential meiotic structure-specific endonuclease 1 (EME1) resolvase complex. Like in the *Cdk2*^*Y15S*^ model, *Mus81*^*−/−*^ spermatocytes show a significant increase in the number of MLH1 foci. Despite this increase in MLH1 foci, this did not translate to a greater number of overall chiasmata in the *Cdk2*^*Y15S*^ mice [95]. It was suggested that upon loss of the “back-up” class II CO pathway mediated by MUS81, increased MLH1 foci ensure that the overall numbers of meiotic COs remain the same. Interestingly, the residual COs seen on a *Mlh3*^*−/−*^ background can be further reduced by the additional deletion of *Mus81* [95]. The small amount of remaining COs observed even in this double-mutant mouse is indicative of additional pathways mediating CO formation. This could perhaps be through other resolvase complexes such as structure-specific endonuclease subunit SLX1 (SLX1)–BTBD12 domain-containing protein 12 (BTBD12) or flap endonuclease GEN homolog 1 (GEN1) [96–98]. Indeed, an almost identical increase in MLH1 foci formation can be observed upon deletion of BTBD12. It is thought that SLX1–BTBD12 might drive recombination intermediates towards class II CO events, thereby promoting MUS81-mediated CO [99]. In summary, this may suggest that CDK2 might not regulate CO designation but might be involved in maturation of designated sites. Interestingly, the budding yeast counterparts of both EME1 (CO junction endonuclease MMS4 [mms4]) and BTBD12 (Slx4) have previously been shown to be targets of CDK-associated activity [100–102]. The implications of whether these resolvases might also be regulated by CDK activity during mammalian meiosis are still poorly understood. If phosphorylation of such complexes by hyperactive CDK2^Y15S^ led to the inhibition of the class II CO pathways, this might lead to a compensatory increase in the numbers of MLH1 foci, similar to that seen upon deletion of *Mus81* or *Btbd12*.

### Why does T-loop mutagenesis of CDK2 prevent the repair of early recombination intermediates?

The failed repair of early recombination intermediates in *Cdk2*^*T160A*^ spermatocytes suggests that CDK2 might be involved in the process of DNA damage repair. It is known that *Cdk2*^*−/−*^ mouse embryonic fibroblasts (MEFs) exhibit delayed resumption of DNA replication following DNA damage induced by irradiation. Similarly, *Cdk2*^*−/−*^ mice display increased sensitivity in response to irradiation and die earlier than WT controls [103]. When investigated in greater detail, it was found that γH2AX foci form at lower levels in response to DNA damage in the absence of CDK2, suggesting a delayed DNA damage response (reviewed in [104]). Interestingly, the early stages of the DNA damage response activated by meiotic DSBs in *Cdk2*^*T160A*^ spermatocytes were not notably different from those seen in WT spermatocytes. We observed typical γH2AX formation during leptonema, and the levels of γH2AX on autosomal chromatin were properly resolved upon completion of synapsis. RPA, RNF212, and MSH4 foci also formed at sites of recombination at levels similar to those of WT spermatocytes. Despite this, the behavior and appearance of early-recombination-nodule–associated DNA damage proteins on fully synapsed homolog axes were different from WT. Early recombination nodules were inappropriately stabilized in a manner that prevented their removal from SC axes. A simple explanation for this would be that the structure of the SC in *Cdk2*^*T160A*^ is less permissive towards the repair of early recombination intermediates because of the loss of CDK2-mediated phosphorylation of one or more axis-associated proteins. A similar concept has been observed in *Caenorhabditis elegans*, in which structural changes to the SC are proposed to “compartmentalize” recombination intermediates destined to become COs in a manner that preferentially promotes their stability over other sites of recombination destined to become NCOs [105–107]. Another explanation would be that T-loop mutagenesis of CDK2 prevents the formation of active CDK2 complexes. This, in turn, prevents the phosphorylation of (unknown) proteins that allow the eventual designation of meiotic recombination intermediates to form LRNs.

### Conclusions

Here, we describe in detail the necessity of the CDK2-activating residue Thr160, firstly for normal maintenance of synapsis and telomere stability during meiotic prophase and secondly for the formation of meiotic COs from recombination intermediates. This work illustrates the importance for the normal regulation of CDK2 activity during meiotic prophase and adds yet further novel, to our knowledge, functions of CDK2 in addition to those currently established. These include promoting homolog synapsis through the regulation of telomere–nuclear envelope interactions [10, 23], regulating meiotic transcription [18], and maintaining homeostasis and differentiation status of spermatogonial stem cells [17]. We conclude that CDK2-associated activity is required at multiple stages during the development of germ cells and that this is enabled by the association of CDK2 with multiple activating partner proteins beyond only the currently known Speedy A. Further research is still needed to uncover the exact substrates that enable CDK2 to act as a pro-CO factor, and these will likely be uncovered through the identification of the specific CDK2-associated factor(s) that localize to LRNs.

## Materials and methods

### Ethics statement

All experimental protocols were approved by the Animal Care and Use Committee of Biological Resource Centre at Biopolis, A*STAR, Singapore (protocol #171268) or under protocol (2004–0038) approved by the Cornell University Animal Care and Use Committee.

### Transgenic mouse lines used in this study

*Cdk2*^*T160A*^ [42], *Speedy A*^*−/−*^ [24], and *Cdk2*^*−/−*^ [28] mice have been previously described and were maintained on a C57BL/6 background. *Cdk2*^*Y15S*^ [15] and *Cdk2*^*Y15S/T160A*^ [17] mice have been previously described and were maintained on a mixed genetic background (FVB/NJ and B6[Cg]-Tyrc-2J/J). Mice were housed under standard conditions, were maintained on a 12-hour light/dark cycle, were fed a standard chow diet containing 6% crude fat, and were treated humanely in compliance with the institutional guidelines for animal care and use.

### Testes histology, immunohistochemistry, and immunofluorescent staining

For histological analyses, testes were fixed for 16 hours at 4°C in modified Davidson’s fixative (8.2% formalin, 33% ethanol, 11% glacial acetic acid in water). Fixed tissues were paraffin-embedded, sectioned at 5 μm, and then stained with the ApopTag Plus Peroxidase In Situ Apoptosis Kit (Sigma-Aldrich: S7101; St. Louis, MO, USA) or HE were used as previously described [18]. For immunohistochemical staining of testis sections or immunofluorescent staining of meiotic chromosome spreads, slides were blocked in PBS containing 0.15% Triton X-100, 10% BSA, 3% skim milk powder for 1 hour at RT, followed by incubation with primary antibodies for 16 hours at 4°C and detection by secondary antibodies (see antibody details in S1 Table).

### Preparation of meiotic chromosome spreads from mouse testes

Seminiferous tubules were extracted from mouse testes of various ages into a solution of 2.2% trisodium citrate solution (75 mM). Seminiferous tubules were then transferred into a hypotonic solution buffer (30 mM Tris-HCl [pH 8.8], 5 mM EDTA, 17 mM trisodium citrate dihydrate, and 50 mM sucrose) for 20 min at room temperature. Seminiferous tubules were then transferred into a solution of 100 mM sucrose solution (pH 8.2). Cells were extracted from tubules by first chopping tubules with a razor blade and then repeatedly passing pipetting chopped tubules through a 200-μl tip (30×). The now cloudy sucrose solution was then separated from the cut tubules and passed through a 100-μm nylon mesh cell strainer (2×). This solution was dropped from a height of 5 cm onto Superfrost Plus Microscope slides (4951PLUS4; Thermo Fisher Scientific, Waltham, MA, USA) coated with 1% paraformaldehyde solution, 0.33% Triton X-100 [pH 9.2] in water). Chromosome spreads were then placed into a humidified box kept at 40°C–50°C for at least 2 hours (maximally 16 h). After this incubation period, the lid to the humidified box was removed, and slides were allowed to partially dry by fanning for approximately 1–2 min on a benchtop. Slides were then moved to −80°C for (indefinite) storage until staining. All staging was performed based upon typical staging guidelines for the XY body during meiotic prophase I [108, 109] or by utilizing the mid-pachytene–specific antibodies against histone H1t.

### Preparation and Giemsa staining of diakinesis spreads from mouse testes

Diakinesis chromosome preparations were prepared as described previously [110]. Briefly, testes were minced in hypotonic buffer (1% sodium citrate). The large pieces of tubules were removed, and cells were further incubated at room temperature for 15 min in the same hypotonic buffer. This was followed by centrifugation and removal of supernatant and fixation of cells in a methanol/acetic acid/chloroform fixative. Following several washes with the fixative, fixed cells were dropped onto slides, quickly dried, and stained with Giemsa. Giemsa staining was performed as previously described [110]. Diakinesis spreads were blocked and stained as described above for testis sections and meiotic chromosome spread preparations.

### Fluorescence imaging

All fluorescence microscopy images were taken using a Zeiss AxioImager Z1 (electron beam lithography) motorized microscope (Carl Zeiss, Oberkochen, Germany). 100× magnification images were taken using an oil immersion Plan Apochromat lens with a 1.4 numerical aperture. All images were taken at RT using Immersol immersion oil (Carl Zeiss) as the imaging medium. Specific signal of primary antibodies was detected using Alexa Fluor secondary antibodies conjugated to Alexa Fluor 488, 500, or 647 fluorophore dyes (Thermo Fisher Scientific). All primary and secondary antibodies used are listed in S1 Table. Images were taken using an Axiocam Hrc camera (Carl Zeiss) using X-cite metal halide as a fluorescence source. Images were acquired using the Zen 2.3 (blue edition) acquisition software. After imaging, further processing was performed using Adobe Photoshop CC 2018 to add pseudocolors and to overlay different channels of costained images. When comparisons were drawn between fluorescent images, lamp intensity and exposure time were kept identical when taking images.

### Focus quantification and focus intensity quantification

Fiji-ImageJ was used to perform both manual and semiautomatic counting of immunofluorescent foci [111]. Semiautomated counts were performed images obtained from the individual channels for each specific immunofluorescent stain, where the threshold was set above background level. Counts were obtained after performing “Watershed” by the “Analyze Particles” functionality. Sizes were manually set from 1.5 to infinity. This method has been previously used to quantify RAD51 foci [112]. If foci were too closely spaced or difficult to calculate accurately using this method, manual particle counting using the “multi-point tool” was used to generate focus counts.

Photoshop CC 2020 was used for semiautomated calculation of focus intensity quantification for telomeric CDK2 or Speedy A signal intensity. The elliptical marquee tool was set to a fixed size of 8 × 8 pixels and was used to highlight individual telomeric foci. The “record measurements” function of the measurement log was then used to record the integrated density value. This same procedure was performed for 4 negative areas of the specific nucleus these values were taken from to generate a background value. This value was averaged and subsequently misused from the telomeric intensity values to normalize them to the background of the cell.

### Calculation of interfocus distances of MLH1

Distances between MLH1 foci were calculated using Fiji software. The measure tool for the segmented line on ImageJ was used to generate distance data.

### Brightfield imaging for HE, TUNEL, and diakinesis chromosome spread preparations

All brightfield images were taken using an Olympus BX-51 motorized microscope (Olympus, Tokyo, Japan). The 40× images were taken using an oil immersion Plan fluor lens with a 0.75× numerical aperture. The 60× images were taken using an oil immersion Plan fluor lens with a 1.25× numerical aperture. All images were taken at RT using Olympus immersion oil as the imaging medium. Images were taken using an Olympus D22 camera using a HAL 100 light source. Images were acquired using CellSens acquisition software. No further processing was performed for brightfield images.

### Statistical analysis

All experiments were repeated at least 3 times or on 3 biological replicates. For immunofluorescence staining, at least 20 images were generated for each biological replicate for each stage presented in this manuscript. Exact N numbers for each experiment or analysis are detailed in figure legends pertaining to that data. GraphPad Prism version 6 (GraphPad Software, La Jolla, CA, USA) was used for all statistical tests, and differences were considered significant when *P* < 0.05.

## Supporting information

S1 Data In separate sheets, the excel spreadsheet contains the numerical data and statistical analysis for Figs 1N, 1O, 1P, 1Q, 2F, 3M, 3N, 3O, 4L, 4X, 5I, 5J, 6G, 7G, 7H, 7I, 7J, 7L, S1A, S2D, S3G, S3H, S3I, S4L, S5J, S5K and S5L. (XLSX)

S1 Table Antibodies used in this study. All primary and secondary antibodies used in this study are listed, including the dilution used, the source, and the catalog number.
(XLSX)

S1 Fig Histological comparisons of WT, *Cdk2*^*T160A*^, and *Cdk2*^*−/−*^ testes. (A) Testis weight plotted as a percentage of bodyweight for WT (orange squares), *Cdk2*^*T160A*^ (blue triangles), and *Cdk2*^*−/−*^ testes (gray diamonds). Only statistical comparisons between *Cdk2*^*T160A*^ and *Cdk2*^*−/−*^ testes are shown; WT is shown for reference. Error bars are indicative of the mean and SD. All data were assumed to be non-normally distributed. Statistical significance between genotypes at each time point was determined by unpaired *t* test. Significance and *P*-values are reported directly over each comparison. Measurements were made from at least 5 biological replicates for the time points shown. The bottom panel is used to show the progression of spermatogenesis of spermatocytes during the first meiotic division. A yellow box is used as a reference for the P30 images shown in panels B–E. Histological sections from WT (B), *Cdk2*^*−/−*^ (C), or *Cdk2*^*T160A*^ (D–E). Suspected apoptotic cells in *Cdk2*^*T160A*^ testes are shown in panel E (yellows arrows) alongside pachytene-stage spermatocytes of healthy morphology (red arrows). The underlying data for (A) can be found in S1 Data. CDK2, cyclin-dependent kinase 2; WT, wild-type.
(TIF)

S2 Fig Comparative analysis of apoptosis in WT, *Cdk2*^*T160A*^, and *Cdk2*^*−/−*^ testes. TUNEL staining of P30 WT (A), *Cdk2*^*T160A*^ (B), and *Cdk2*^*−/−*^ (C) testis sections are shown with hematoxylin nuclear counterstaining. Pachytene-stage spermatocytes of healthy morphology (red P) are shown in A1, B1, and B2. TUNEL-positive cells suspected to be apoptotic pachytene-stage spermatocytes (green P*) are shown in B1 and C1. The supplied key indicates labels for various spermatogenic cell types seen in each image. Panel D shows a comparison of the mean numbers of apoptotic cells counted per tubule in WT (orange squares), *Cdk2*^*T160A*^ (blue triangles), and *Cdk2*^*−/−*^ (gray diamonds) testes. Individual points are representative of at least 200 tubules counted from a single biological replicate. Error bars are indicative of the mean and SD. All data were assumed to be non-normally distributed. Statistical significance between genotypes at each time point was determined by unpaired *t* test. Significance and *P*-values are reported directly over each comparison. The underlying data for (D) can be found in S1 Data. CDK2, cyclin-dependent kinase 2; SD, standard deviation; WT, wild-type.
(TIF)

S3 Fig CDK2/Speedy A localization in *Cdk2*^*−/−*^ and *Speedy A*^*−/−*^ spermatocytes. Chromosome spread preparations from adult (postnatal day 40) testes immunostained with CDK2 (green) and Speedy A (red) in conjunction with SYCP3 (blue) are shown for *Cdk2*^*−/−*^ (A–C) and *Speedy A*^*−/−*^ (D–F) spermatocytes for selected stages of meiotic prophase I. Meiotic arrest occurs at a pachytene-like stage, and CDK2 and Speedy A foci are absent from all stages of meiotic prophase in *Cdk2*^*−/−*^ and *Speedy A*^*−/−*^ spermatocytes. (G) Individual CDK2 and Speedy A focus intensity/telomere was quantified specifically for mid-pachytene stages of WT and *Cdk2*^*T160A*^ spermatocytes. The individual telomeric intensity of all telomeric CDK2 or Speedy A signals was converted into a percentage of the average telomeric signal taken from a single nucleus. Data are presented as individual percentage intensity values for each telomere quantified from 26 nuclei (494 in total) for both CDK2 and Speedy A. WT data are shown using orange bars, and *Cdk2*^*T160A*^ data are shown using blue bars. Highlighted by dashed lines are intervals in which telomeres were found to have a 50%–74% decrease in signal and a 75%–100% decrease in signal. These values were used to plot graphs in Fig 3O and S3I Fig. (H) The average telomeric Speedy A focus intensity/nuclei was quantified specifically for mid-pachytene stages. Data are presented as a mean intensity in AUs ± SD determined from 3 biological replicates (N = 48 overall nuclei counted for WT [orange bars] and N = 48 overall nuclei counted [blue bars] for *Cdk2*^*T160A*^). Telomeric signals were only counted from autosomes and were excluded if involved in telomeric fusion events. All intensity values calculated for a single nucleus were normalized to the background intensity of that nuclei. Percentages of mid-pachytene–stage nuclei with at least one telomere showing a decrease in telomeric Speedy A intensity of ≥50% (as compared to the average telomeric Speedy A intensity for that cell) are quantified in panel I. All data in panels G–I were assumed to be non-normally distributed. Error bars are indicative of the mean and SD. Statistical significance between genotypes was determined by unpaired *t* test. Significance and *P*-values are reported directly over each comparison. The underlying data for (G, H, I) can be found in S1 Data. AU, arbitrary unit; CDK2, cyclin-dependent kinase 2; SD, standard deviation; SYCP, synaptonemal complex protein; WT, wild-type.
(TIF)

S4 Fig Comparison of RPA2 dynamics in WT, *Cdk2*^*T160A*^, *and Cdk2*^*−/−*^ spermatocytes. P40 chromosome spread preparations immunostained for RPA2 (green) and SYCP3 (red) are shown for WT (A–D), *Cdk2*^*T160A*^ spermatocytes (E–H), and *Cdk2*^*−/−*^ (I–K) for selected stages of meiotic prophase I. During leptotene and zygotene stages, WT (A–B), *Cdk2*^*T160A*^ (E–F), and *Cdk2*^*−/−*^ (I–J) spermatocytes show RPA2 foci localized to chromosomal axes before full synapsis. During early-pachytene in WT (C) and *Cdk2*^*T160A*^ (G) spermatocytes, RPA2 foci remain localized to paired axes. In *Cdk2*^*−/−*^ spermatocytes only, a pachytene-like arrest state is achieved (K). Here, RPA2 foci are observed to remain bound to stretched chromosomal axes despite extensive nonhomologous synapsis. In WT spermatocytes, SC-associated RPA2 foci decrease in number upon transition to mid-pachytene (D). In mid-pachytene *Cdk2*^*T160A*^ nuclei, RPA2 foci numbers remain high (H). All main images are representative of at least 20 images taken for equivalent stages. Similar staining patterns were confirmed in at least 3 biological replicates. In all images, scale bars are representative of 5 μm. RPA2 foci were quantified specifically for early-pachytene stages and mid-pachytene stages (L) by counting the average numbers of RPA2 foci per nucleus. Data are presented as individual foci counts for WT (orange bars, N = 90 for early-pachytene and mid-pachytene stages), *Cdk2*^*T160A*^ (blue bars, N = 50 for early-pachytene and mid-pachytene stages), and *Cdk2*^*−/−*^ (gray bars, N = 50 for pachytene-like stage). Error bars are indicative of the mean and SD. All data were assumed to be non-normally distributed. Statistical significance between genotypes was determined by unpaired *t* test. Significance and *P*-values are reported directly over each comparison. The underlying data for (L) can be found in S1 Data. CDK2, cyclin-dependent kinase 2; RPA2, replication protein A; SC, synaptonemal complex; SD, standard deviation; SYCP, synaptonemal complex protein; WT, wild-type.
(TIF)

S5 Fig Phenotypic analysis of *Cdk2*^*Y15S*^ and *Cdk2*^*Y15S/T160A*^ spermatocytes in the first wave of spermatogenesis. (A–I) P25 chromosome spread preparations of spermatocytes from WT (A, D, G), *Cdk2*^*Y15S*^ (B, E, H), and *Cdk2*^*Y15S/T160A*^ (C, F, I) immunostained for the indicated proteins. Similar staining patterns were confirmed in at least 3 biological replicates. In all main images, scale bars are representative of 5 μm; in all inset pictures, scale bars are representative of 1.25 μm. (J) Quantification of incompletely synapsed bivalents. (K) Quantification of nonsynaptic end-to-end associations of homologs. (L) Quantification of autosomal RAD51 foci in mid-pachytene–like cells. All *p*-values are calculated from Student two-tailed *t* test. Statistics Box Height = data points between the first and third quartiles of the distribution, whiskers = minimum and maximum values, “+” represents mean value. The underlying data for (J, K, L) can be found in S1 Data. CDK2, cyclin-dependent kinase 2; RAD51, RAD51 recombinase; WT, wild-type.
(TIF)

## Abbreviations

ACA: autocentromere antibody
A*STAR: Agency for Science, Technology, and Research
BTBD12: BTBD12 domain-containing protein 12
CDK2: cyclin-dependent kinase 2
*Cntd1*: cyclin N-terminal domain-containing protein 1
CO: crossover
C–C: centromeric end–centromeric end
DMC1: dosage suppressor of Mck1 homolog
DSB: double-strand break
EME1: essential meiotic structure-specific endonuclease 1
GEN1: flap endonuclease GEN homolog 1
HE: hematoxylin–eosin
*Hei10*: human enhancer of invasion clone 10
H3K9me3: trimethylated lysine 9 of histone H3
IMCB: Institute of Molecular and Cellular Biology
JAX: The Jackson Laboratory
LRN: late recombination nodule
MEF: mouse embryonic fibroblast
MLH1: MutL homolog 1
mms4: crossover junction endonuclease MMS4
MSCI: meiotic sex chromosome inactivation
MSH4/5: MutS protein homolog 4/5
MUS81: crossover junction endonuclease MUS81
NCO: noncrossover
NC–NC: noncentromeric end–noncentromeric end
NUS: National University of Singapore
PAR: pseudoautosomal region
*Prr19*: proline-rich protein 19
RAD51: RAD51 recombinase
RAP1: TERF2 interacting protein
RINGO: rapid inducer of G2/M progression in oocytes
*Rnf212*: ring finger protein 212
RPA: replication protein A complex
RPA2: replication protein A 32 kDa subunit
SC: synaptonemal complex
SD: standard deviation
SLX1: structure-specific endonuclease subunit SLX1
SPO11: meiotic recombination protein SPO11
SUMO: small ubiquitin-like modifier
SYCP: synapotonemal complex protein
WEE1: Wee1-like protein kinase
WT: wild-type
γH2AX: phosphoserine 139 histone H2A variant
