# Supplementary figures and images for: A novel function for CDK2 activity at meiotic crossover sites
Source: PLoS Biol. 2020 Oct 19;18(10):e3000903. doi: 10.1371/journal.pbio.3000903 (PMC7595640; doi:10.1371/journal.pbio.3000903)

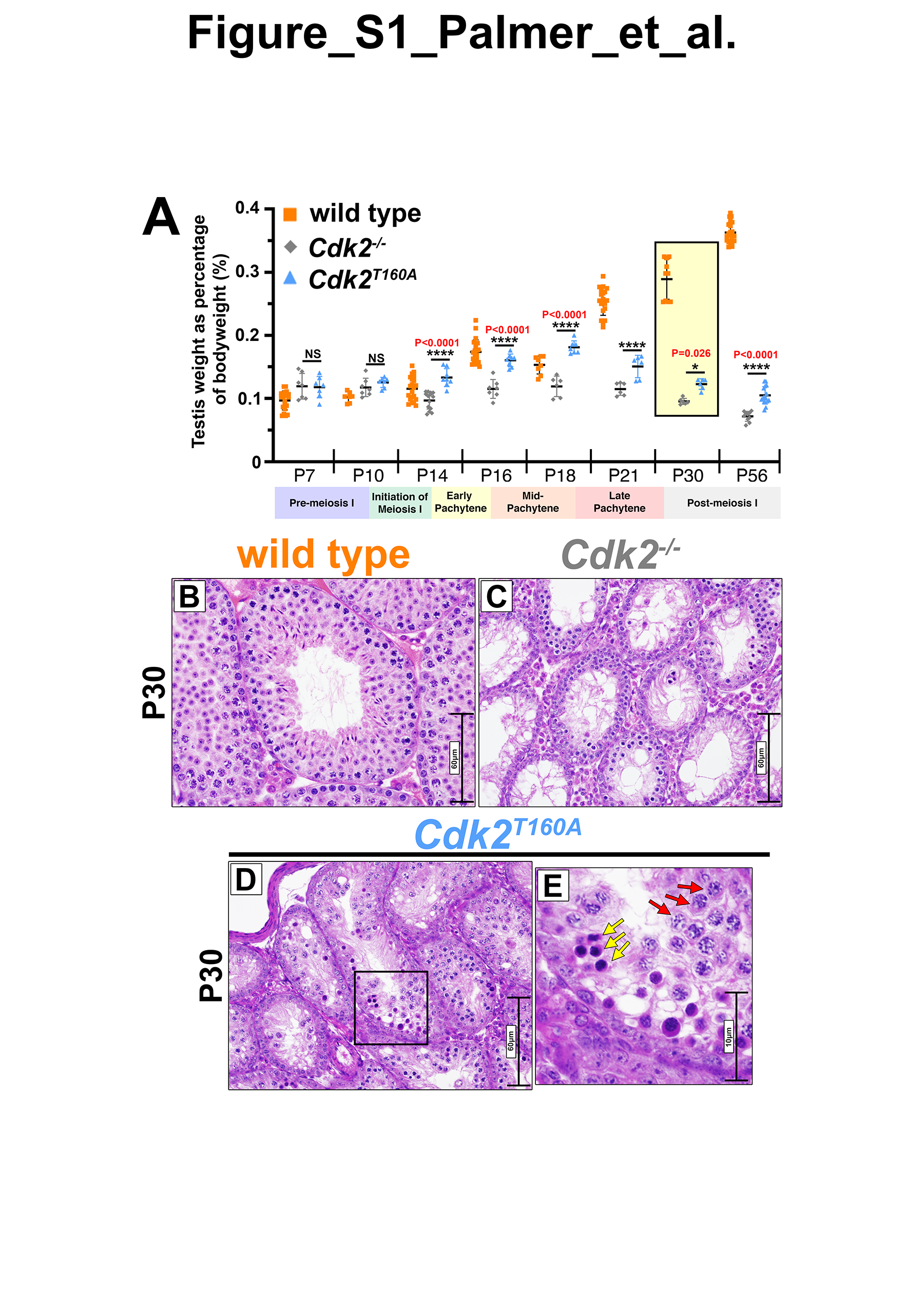

Supplement: S1 Fig — (A) Testis weight plotted as a percentage of bodyweight for WT (orange squares), Cdk2T160A (blue triangles), and Cdk2−/− testes (gray diamonds). Only statistical comparisons between Cdk2T160A and Cdk2−/− testes are shown; WT is shown for reference. Error bars are indicative of the mean and SD. All data were assumed to be non-normally distributed. Statistical significance between genotypes at each time point was determined by unpaired t test. Significance and P-values are reported directly over each comparison. Measurements were made from at least 5 biological replicates for the time points shown. The bottom panel is used to show the progression of spermatogenesis of spermatocytes during the first meiotic division. A yellow box is used as a reference for the P30 images shown in panels B–E. Histological sections from WT (B), Cdk2−/− (C), or Cdk2T160A (D–E). Suspected apoptotic cells in Cdk2T160A testes are shown in panel E (yellows arrows) alongside pachytene-stage spermatocytes of healthy morphology (red arrows). The underlying data for (A) can be found in S1 Data. CDK2, cyclin-dependent kinase 2; WT, wild-type. (TIF) [file pbio.3000903.s003.tif]

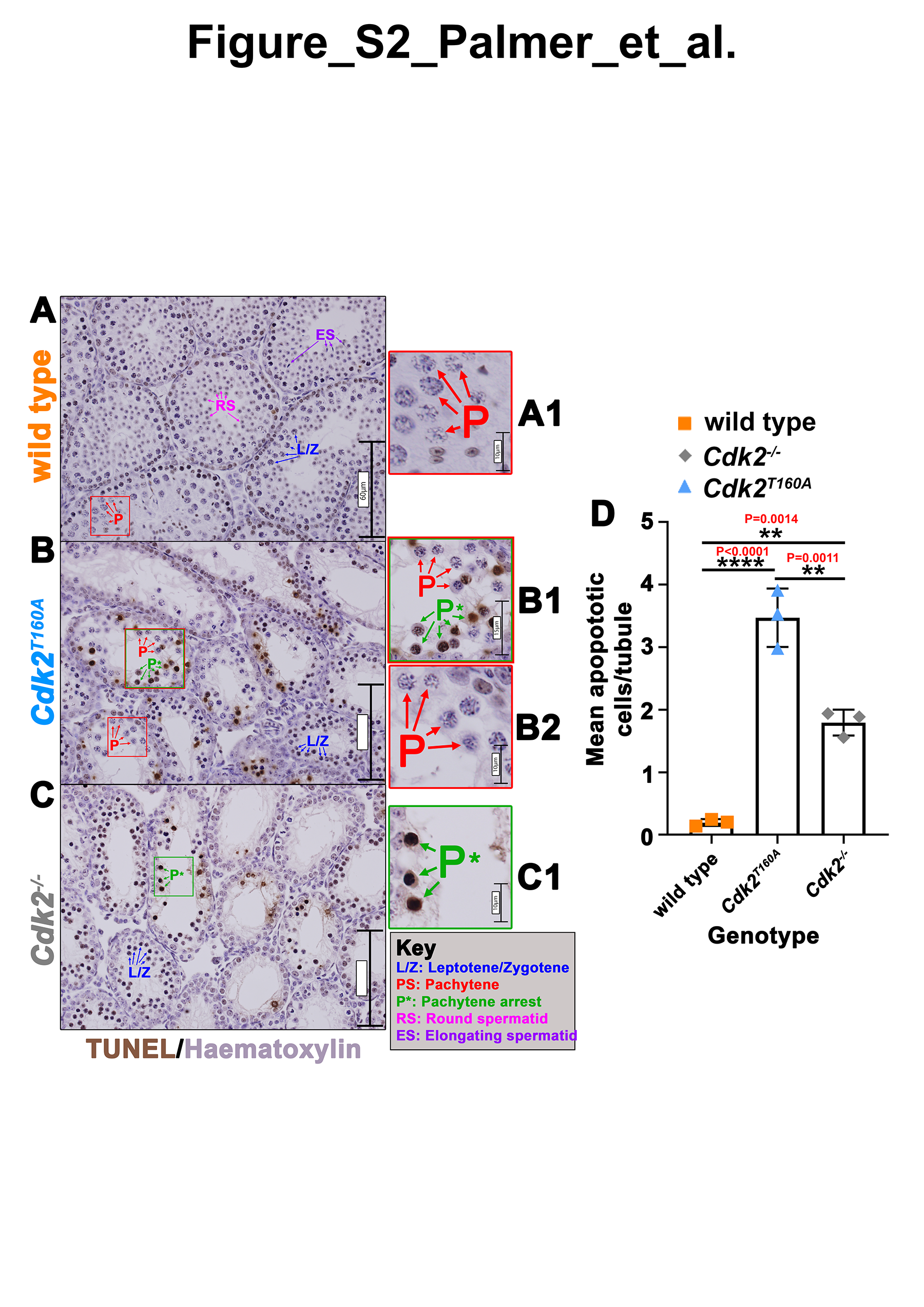

Supplement: S2 Fig — TUNEL staining of P30 WT (A), Cdk2T160A (B), and Cdk2−/− (C) testis sections are shown with hematoxylin nuclear counterstaining. Pachytene-stage spermatocytes of healthy morphology (red P) are shown in A1, B1, and B2. TUNEL-positive cells suspected to be apoptotic pachytene-stage spermatocytes (green P*) are shown in B1 and C1. The supplied key indicates labels for various spermatogenic cell types seen in each image. Panel D shows a comparison of the mean numbers of apoptotic cells counted per tubule in WT (orange squares), Cdk2T160A (blue triangles), and Cdk2−/− (gray diamonds) testes. Individual points are representative of at least 200 tubules counted from a single biological replicate. Error bars are indicative of the mean and SD. All data were assumed to be non-normally distributed. Statistical significance between genotypes at each time point was determined by unpaired t test. Significance and P-values are reported directly over each comparison. The underlying data for (D) can be found in S1 Data. CDK2, cyclin-dependent kinase 2; SD, standard deviation; WT, wild-type. (TIF) [file pbio.3000903.s004.tif]

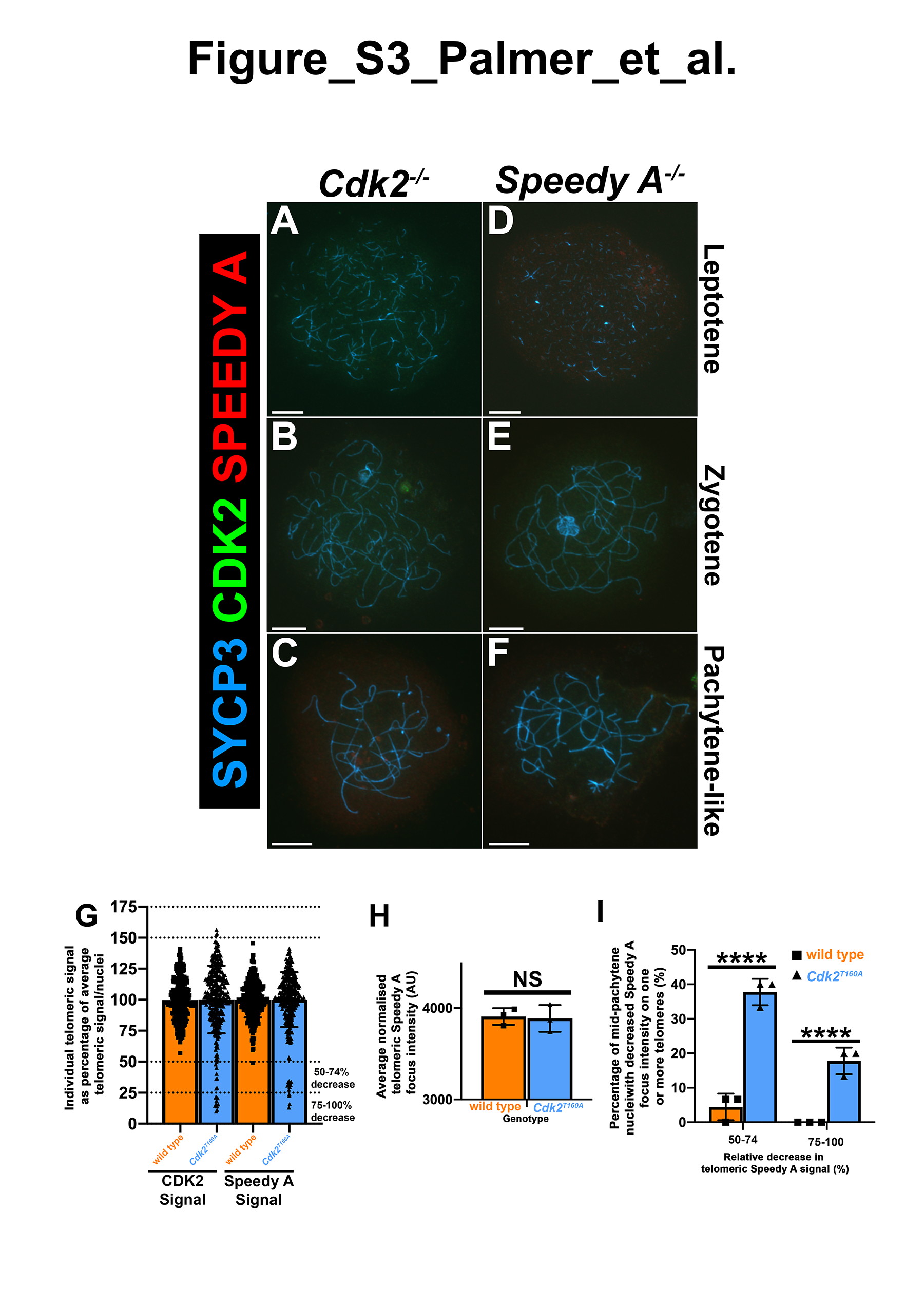

Supplement: S3 Fig — Chromosome spread preparations from adult (postnatal day 40) testes immunostained with CDK2 (green) and Speedy A (red) in conjunction with SYCP3 (blue) are shown for Cdk2−/− (A–C) and Speedy A−/− (D–F) spermatocytes for selected stages of meiotic prophase I. Meiotic arrest occurs at a pachytene-like stage, and CDK2 and Speedy A foci are absent from all stages of meiotic prophase in Cdk2−/− and Speedy A−/− spermatocytes. (G) Individual CDK2 and Speedy A focus intensity/telomere was quantified specifically for mid-pachytene stages of WT and Cdk2T160A spermatocytes. The individual telomeric intensity of all telomeric CDK2 or Speedy A signals was converted into a percentage of the average telomeric signal taken from a single nucleus. Data are presented as individual percentage intensity values for each telomere quantified from 26 nuclei (494 in total) for both CDK2 and Speedy A. WT data are shown using orange bars, and Cdk2T160A data are shown using blue bars. Highlighted by dashed lines are intervals in which telomeres were found to have a 50%–74% decrease in signal and a 75%–100% decrease in signal. These values were used to plot graphs in Fig 3O and S3I Fig. (H) The average telomeric Speedy A focus intensity/nuclei was quantified specifically for mid-pachytene stages. Data are presented as a mean intensity in AUs ± SD determined from 3 biological replicates (N = 48 overall nuclei counted for WT [orange bars] and N = 48 overall nuclei counted [blue bars] for Cdk2T160A). Telomeric signals were only counted from autosomes and were excluded if involved in telomeric fusion events. All intensity values calculated for a single nucleus were normalized to the background intensity of that nuclei. Percentages of mid-pachytene–stage nuclei with at least one telomere showing a decrease in telomeric Speedy A intensity of ≥50% (as compared to the average telomeric Speedy A intensity for that cell) are quantified in panel I. All data in panels G–I were assumed to be non-normally dis [file pbio.3000903.s005.tif]

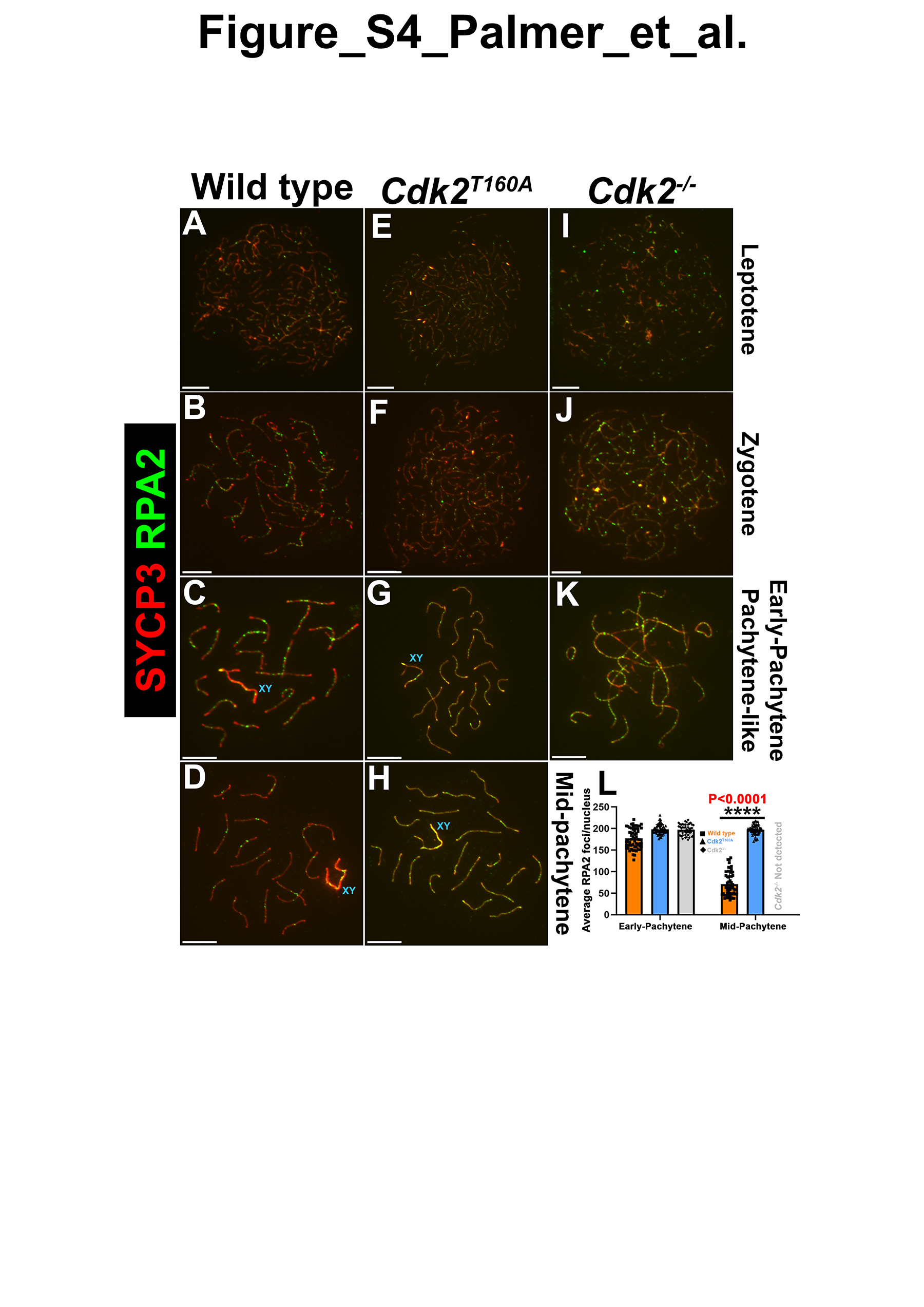

Supplement: S4 Fig — P40 chromosome spread preparations immunostained for RPA2 (green) and SYCP3 (red) are shown for WT (A–D), Cdk2T160A spermatocytes (E–H), and Cdk2−/− (I–K) for selected stages of meiotic prophase I. During leptotene and zygotene stages, WT (A–B), Cdk2T160A (E–F), and Cdk2−/− (I–J) spermatocytes show RPA2 foci localized to chromosomal axes before full synapsis. During early-pachytene in WT (C) and Cdk2T160A (G) spermatocytes, RPA2 foci remain localized to paired axes. In Cdk2−/− spermatocytes only, a pachytene-like arrest state is achieved (K). Here, RPA2 foci are observed to remain bound to stretched chromosomal axes despite extensive nonhomologous synapsis. In WT spermatocytes, SC-associated RPA2 foci decrease in number upon transition to mid-pachytene (D). In mid-pachytene Cdk2T160A nuclei, RPA2 foci numbers remain high (H). All main images are representative of at least 20 images taken for equivalent stages. Similar staining patterns were confirmed in at least 3 biological replicates. In all images, scale bars are representative of 5 μm. RPA2 foci were quantified specifically for early-pachytene stages and mid-pachytene stages (L) by counting the average numbers of RPA2 foci per nucleus. Data are presented as individual foci counts for WT (orange bars, N = 90 for early-pachytene and mid-pachytene stages), Cdk2T160A (blue bars, N = 50 for early-pachytene and mid-pachytene stages), and Cdk2−/− (gray bars, N = 50 for pachytene-like stage). Error bars are indicative of the mean and SD. All data were assumed to be non-normally distributed. Statistical significance between genotypes was determined by unpaired t test. Significance and P-values are reported directly over each comparison. The underlying data for (L) can be found in S1 Data. CDK2, cyclin-dependent kinase 2; RPA2, replication protein A; SC, synaptonemal complex; SD, standard deviation; SYCP, synaptonemal complex protein; WT, wild-type. (TIF) [file pbio.3000903.s006.tif]

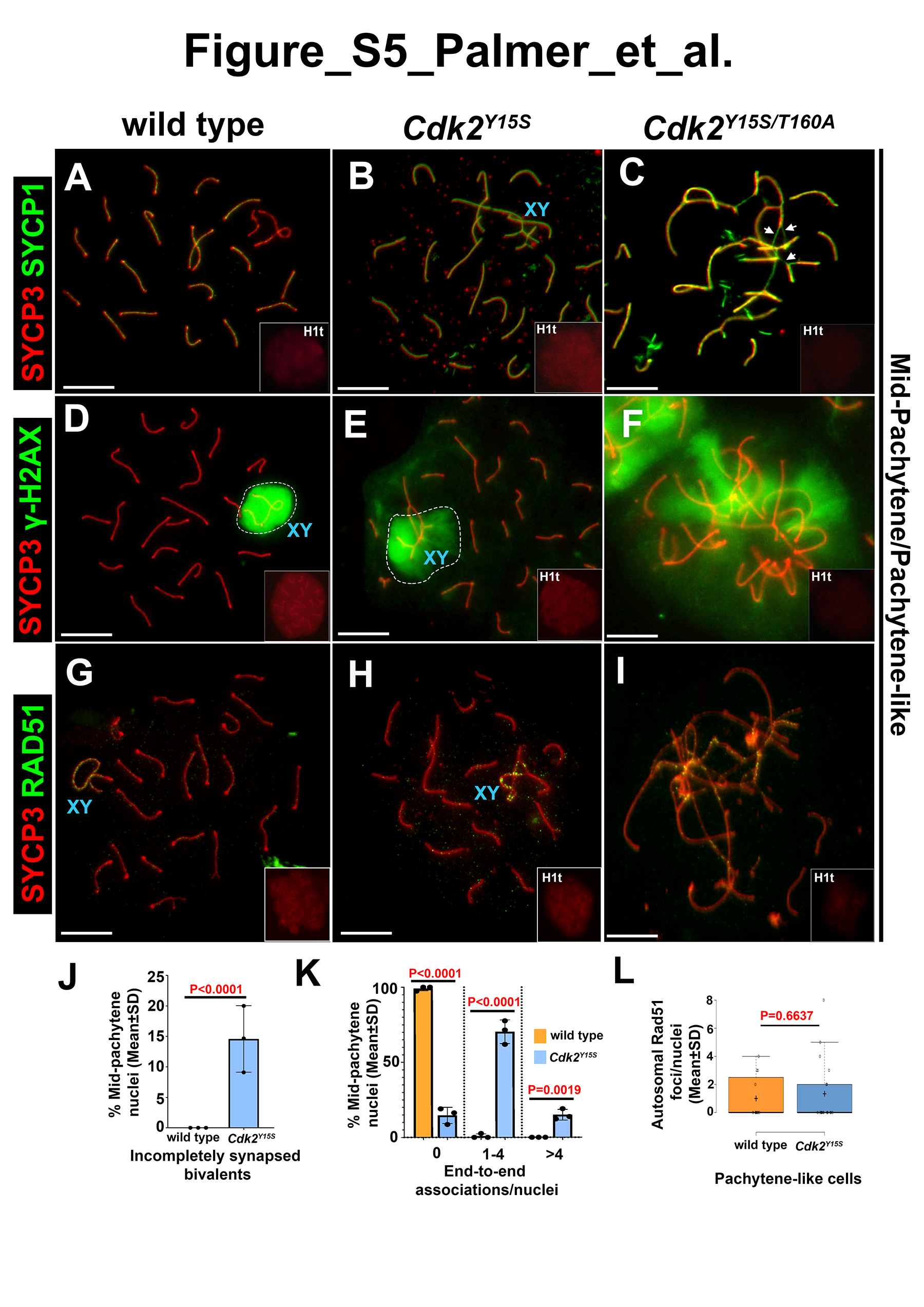

Supplement: S5 Fig — (A–I) P25 chromosome spread preparations of spermatocytes from WT (A, D, G), Cdk2Y15S (B, E, H), and Cdk2Y15S/T160A (C, F, I) immunostained for the indicated proteins. Similar staining patterns were confirmed in at least 3 biological replicates. In all main images, scale bars are representative of 5 μm; in all inset pictures, scale bars are representative of 1.25 μm. (J) Quantification of incompletely synapsed bivalents. (K) Quantification of nonsynaptic end-to-end associations of homologs. (L) Quantification of autosomal RAD51 foci in mid-pachytene–like cells. All p-values are calculated from Student two-tailed t test. Statistics Box Height = data points between the first and third quartiles of the distribution, whiskers = minimum and maximum values, “+” represents mean value. The underlying data for (J, K, L) can be found in S1 Data. CDK2, cyclin-dependent kinase 2; RAD51, RAD51 recombinase; WT, wild-type. (TIF) [file pbio.3000903.s007.tif]
